# Integrins: Moonlighting Proteins in Invadosome Formation

**DOI:** 10.3390/cancers11050615

**Published:** 2019-05-02

**Authors:** Rafael Peláez, Ana Pariente, Álvaro Pérez-Sala, Ignacio M. Larrayoz

**Affiliations:** Biomarkers and Molecular Signaling Group, Neurodegenerative Diseases Area Center for Biomedical Research of La Rioja, CIBIR, c.p., 26006 Logroño, Spain; apariente@riojasalud.es (A.P.); aperez@riojasalud.es (Á.P.-S.)

**Keywords:** integrins, invadopodia, podosome, cancer, signaling, microenvironment, stromal cells, matrix, forces

## Abstract

Invadopodia are actin-rich protrusions developed by transformed cells in 2D/3D environments that are implicated in extracellular matrix (ECM) remodeling and degradation. These structures have an undoubted association with cancer invasion and metastasis because invadopodium formation in vivo is a key step for intra/extravasation of tumor cells. Invadopodia are closely related to other actin-rich structures known as podosomes, which are typical structures of normal cells necessary for different physiological processes during development and organogenesis. Invadopodia and podosomes are included in the general term ‘invadosomes,’ as they both appear as actin puncta on plasma membranes next to extracellular matrix metalloproteinases, although organization, regulation, and function are slightly different. Integrins are transmembrane proteins implicated in cell–cell and cell–matrix interactions and other important processes such as molecular signaling, mechano-transduction, and cell functions, e.g., adhesion, migration, or invasion. It is noteworthy that integrin expression is altered in many tumors, and other pathologies such as cardiovascular or immune dysfunctions. Over the last few years, growing evidence has suggested a role of integrins in the formation of invadopodia. However, their implication in invadopodia formation and adhesion to the ECM is still not well known. This review focuses on the role of integrins in invadopodium formation and provides a general overview of the involvement of these proteins in the mechanisms of metastasis, taking into account classic research through to the latest and most advanced work in the field.

## 1. Introduction

Invasion is a dynamic and complicated process with critical roles in cell survival and the development of multicellular animals. They play roles in such important processes as cell migration and invasion during embryogenesis, wound healing, and immune response, among others [1,2,3]. However, uncontrolled cell migration has been linked to cancer invasion and metastasis [4]. Actin has a key role in cell migration and invasion because is responsible for providing the necessary force for both processes [5]. In the cell, actin is present in filamentous and monomeric forms. Actin filaments are polar structures with an assembling region also known as the “plus” or “barbed” end, and a disassembly region named the “pointed” or “minus” end, both of which change dynamically through processes that require ATP [2]. Myosin and several actin-binding proteins also participate in generating this force through many signal pathways involving plasma membrane phospholipids and the RHO family of small GTPases [4].

## 2. Integrins

Integrins are the main cellular receptors for ECM components, i.e., collagen, laminin, fibronectin, or vitronectin [6,7,8]. They are transmembrane heterodimer receptors consisting of an α and a β subunit. There are 18 distinct α and 8 distinct β subunits for a total of 24 α/β combinations [9]. Integrins are also involved in cell-to-cell interactions, acting as cell receptors [6,8]. Ligand specificity relies primarily on α subunits, while cell signal transduction depends on both α and β subunits. Intracellular integrin domains associate with many cytoskeletal proteins (α-actinin, talin, filamin) and signaling molecules (focal adhesion kinase (FAK), cytohesin-1, β3-endonexin), modulating numerous cellular processes [6].

Glycosylation of integrins is one of the critical posttranslational steps that determinates the fate and function of integrins [8]. In general, this modification consists of the addition of carbohydrates (glycans) to different molecules such as lipids or proteins. Depending on where glycans are attached, integrins can be N-(amino group of asparagine residues) or *O*-(hydroxyl group of threonine or serine residues) glycosylated [10]. Integrin *N*-glycosylation is required for their expression at the plasma membrane, heterodimerization, stabilization, and ligand interaction. Integrin *N*-glycan remodeling by glycotransferases has been implicated in adhesion and migration of tumor cells [8,11,12,13]. Likewise, *O*-glycosylation of integrins has been shown to be involved in both pro- and anti-oncogenic events, depending on the structure of the *O*-glycan [14,15,16]. Moreover, integrins also recognize and bind to glycans of the ECM. Alterations in the integrin glycosylation pattern have been reported to associate with cancer progression [8].

Integrins arrive at the plasma membrane in an inactivated conformation. Internal signals pre-activate the integrin structure, and then ligand binding generates the complete activation condition to induce adhesome formation. The adhesome is the conjunction of signaling, cytoskeletal, and scaffolding proteins that together form the machinery involved in survival, migration, differentiation, and polarity of the cell [17,18,19]. Integrin signaling transduction occurs in a bidirectional way through the plasma membrane (inside-out, outside-in) by conformational changes in the α and β subunits. Upon ligand binding, integrins are connected to the cytoskeleton through recruited adaptors, actin, and signaling proteins [20]. Among the adaptor proteins, talin is one of the most important, as it directly interacts with integrins, favoring the linkage of cytoskeleton and integrin receptors, and plays a key role in inside-out signaling [20].

Integrins are involved in different critical biological processes that determine the fate of the cell, such as adhesion, proliferation, survival, and migration [6,8,17,19,20]. Integrin mutations, silencing, or over-expression/activation are usually associated with embryo death or severe diseases like inflammatory bowel diseases, multiple sclerosis and other autoimmune diseases, pulmonary and hepatic fibrosis, immunodeficiency diseases, allergen-induced airway responses and airway inflammation, and stroke and ischemic heart diseases [21,22,23,24], and anti-integrin directed therapies such as vedolizumab, natalizumab, intrifiban, tirofiban, or abciximab are now being applied for these diseases in clinical settings [22,25]. However, their deregulation is also liked to cancer development and progression [26,27,28]. Adhesion mediated by integrins takes place via focal adhesion. This signaling pathway includes integrin-linked protein kinase (ILK), FAK, phospholipase C and the proteins of the RHO family [6]. A correct integrin activation by ECM binding and cell membrane signaling is critical for cell proliferation, survival and migration. Integrins participate in the regulation of G1 checkpoint via different signaling processes that activate FAK, extracellular signal–regulated kinase (ERK), mitogen-activated protein kinase (MAPK), and the RHO GTPase family [6,19,29,30]. Moreover, they are involved in mitosis, as loss of integrins provokes aberrant cellular division [31]. Integrins induce the protrusion of the cell membrane through the RAC, ILK, and FAK pathways to form cell structures of adhesion and migration [6,17]. Finally, cell adhesion machinery also protects the cell against cell death and promotes survival as integrins activate PI3K/AKT and ERK pathways [6,30].

## 3. Invadosomes

There are different types of actin filament structures: lamellipodia, filopodia, stress fibers, and invadosomes. Lamellipodia are thin, branched structures located at the leading edge of migrating cells that constantly extend and contract themselves, forming membrane ruffles contributing to cell movement. Filopodia are thin, finger-like protrusions made of parallel actin filament bundles involved in sensory tasks. Stress fibers are special actin filament structures with the ability to respond to ECM stiffness [2,32]. Invadosomes are a singular type of actin filament with the ability to degrade the ECM through different metalloproteinases [33]. Invadosomes were first observed in 1980 as circular rosettes in Rous Sarcoma Virus transformed fibroblasts [34]. They appear as micro-domains in the ventral surface of the cell and have different conformations (individual dots, rosettes, aggregates and/or linear structures). They have been shown to act as matrix mechanosensors, sensing and responding to changes in the ECM stiffness. It has been proposed that there is a common precursor for the different invadosome conformations, and, depending on the stimulus received, one conformation or another is adopted [35].

There are two types of invadosome: podosomes and invadopodia. Both are formed from a similar actin-rich core surrounded by different scaffolding, adhesion, and signaling actin-associated proteins [36]. Despite their structural similarities, the term podosome usually refers to invadosomes formed in non-malignant cells, such as macrophages, dendritic cells, neutrophils, osteoclasts, and endothelial cells [37,38], whereas the term invadopodium is usually applied to cancer cells. Moreover, podosomes are shorter, more abundant, and less protrusive than invadopodia, and, furthermore, they are only stable for minutes, while invadopodia have a lifespan of hours, although some molecular differences have been also observed (Table 1) [2,3,39,40].

**Figure 1:**
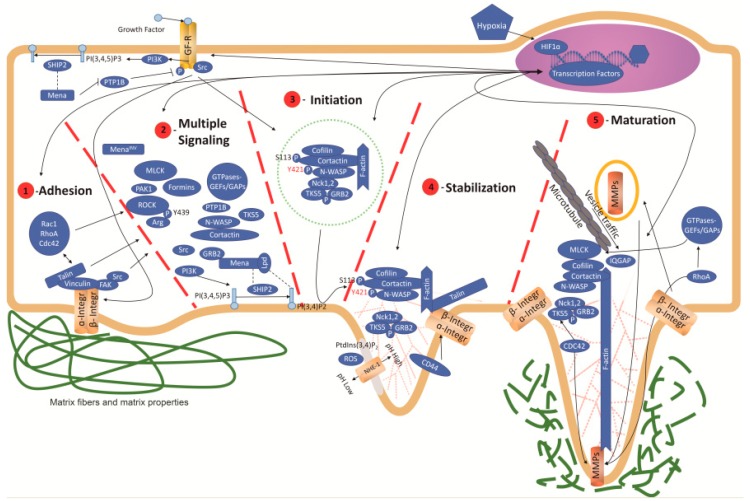
Schematic representation of invadosome formation after cellular stimuli reception. Integrin and/or other membrane receptors (1) generate, after cell adhesion or stimulation, signaling cues (2) that recruit the actin polymerization-branching machinery to plasma membrane domains (3). Actin polymerization and molecular signals stabilize invadosome-growing protrusion in plasma membrane (4), and MMPs-dependent matrix degradation constitutes the final stage (5).

Invadosome formation is stimulated by growth factors (e.g., EGF, TGF-β and PDGF) inducing phosphorylation and activation of many key protein regulators of invadosome formation [41,42,43,44]. At the same time, invadosomes interact with the ECM through integrins and other receptors, such as the hyaluronic receptor (CD44) and the discoidin domain receptors (DDRs), to transmit extracellular and intracellular signals (Figure 1) [45,46,47,48]. In this review, we will see that integrins, and also other ECM adhesion receptors, seem to be implicated in many of the processes and stages of invadosome formation, from the initial steps of anchoring and protein recruitment to the final steps of matrix degradation or structure dissembling.

Literature describes that invadopodium formation has 3 stages: initiation, stabilization, and maturation (Figure 1) [49]. Focal adhesion structures (FA) established between the cell and the ECM need to be dissociated by FAK and SRC kinase in order to allow invadosome formation. SRC kinase and protein kinase C (PKC) then stimulate the primary actin nucleation by phosphorylation of several key regulators, including the F-actin binding protein cortactin, 110 kDa actin filament associated protein (AFAP110), and fascin [42]. It has been proposed that FA are precursors or storehouses for invadopodium and podosome formation [50]. Cell adhesion and/or growth factor signaling, through FAK, SRC, PI3K and many other cascades, stabilizes and promotes invadosome structure (Figure 1) [39]. Vinculin or talin are direct linkers of integrins to the actin cytoskeleton [51], and together with α-actinin, paxilin, and zyxin, are the main components responsible for early sensing and mechano-transduction. In living cells, local interactions between myosin II with actin and motor proteins seems to be critical in order to supply global order to podosomes [52], although SRC is necessary and sufficient for podosome induction in fibroblasts [36]. SRC also activates synaptojanin 2 to transform phosphatidylinositol-trisphosphate (PIP3) into phosphatidylinositol-bisphosphate (PIP2), which interacts with tyrosine kinase substrate with five SH3 domains (TKS5) in the membrane initiation point. Finally, SRC phosphorylates cortactin to recruit NCK adaptor protein 1 (NCK1) and transfer the signaling, through cell division control protein 42 (CDC42) and Wiskott–Aldrich syndrome protein (N-WASP) [53]. CDC42 and transducer of Cdc42-dependent actin assembly protein 1 (TOCA1), an Fes/CIP4 homology-Bin/Amphiphysin/Rvs (F-BAR) family member of plasmatic membrane curvature proteins, also activate the N-WASP/WIP complex to promote actin polymerization [54].

Invadosomes are composed of several actin networks and different actin crosslinking proteins (α-actinin, fascin, fimbrin, supervillin). Together they form actin-rich microdomains and provide the mechanically stable polymerized F-actin necessary to hold up the invadosome structure [40]. Invadosome structure formation initiates with the arrangement of an actin–cortactin nucleus surrounded by different actin regulatory proteins, such as the actin related protein 2/3 complex (ARP2/3), N-WASP, or cofilin (Figure 1) [55,56]. It has been proposed that both N-WASP and cortactin act in a synergetic way in order to activate the ARP2/3 complex [57], as well as to recruit cofilin and gelsolin, among other actin dynamic regulators [40]. TKS5 links the actin complex to plasma membrane phosphadidylinositol 3,4-bisphosphate (PI(3,4)P2) and recruits moesin-NHE1 to increase local pH. Afterwards, cofilin is activated to induce actin polymerization and cortactin phosphorylation at the end of the stabilization stage [39]. This signal is amplified to increase the actin structure before maturation (Figure 1) [39]. Cortactin phosphorylation stimulates the formation of the complex between ARP2/3 and N-WASP, which in turn associates with CDC42, WASP-interacting protein (WIP), and dynamin [3]. In addition, TKS5 is able to bind, direct or indirectly, to growth factor receptor bound protein 2 (GRB2), N-WASP, and NCK1 andNCK2 (Figure 1) [39]. Once assembled, invadosomes promote ECM degradation by secretion of several matrix metalloproteinases: MMP2, MMP9, a desintegrin and metalloproteinase (ADAM), and membrane type 1 matrix metalloproteinase (MT1-MMP) [58]. Localization and secretion of MMPs is regulated in invadopodia by TKS4 and cortactin, respectively, and both of them have a regulator role in podosome and invadopodium maturation (Figure 1) [59].

Invadopodia contain many lipid rafts, indicating that molecular clustering, formation of signaling complexes, and molecular recruitment are necessary for invadopodium formation. Cholesterol depletion, which disrupts lipid raft formation, blocks invadopodium degradative activity [60]. Furthermore, vesicle traffic and molecular recycling are very important factors to the modulation of invadopodium development and activity [36,53,61].

Moreover, other mechanisms of regulation have been reported. Thus, miRNA143 and miRNA145 can negatively regulate podosome formation, although their role in invadopodium formation has not been established [44]. In addition, NOX activation and reactive oxygen species (ROS) induction can regulate podosome and invadopodium formation [62]. Another protein that participates in invadopodium regulation is the cancer-related isoform of mammalian enabled protein (MENA^inv^). MENA^inv^ seems to induce invadopodium activity, enhancing its sensitivity to EGF as well as the phosphorylated status of cortactin. As a result, the invasiveness of cancer cells is increased [63].

Recently, a new invadosome type has been described in endothelial, embryonic, and tumor cells. It is a structure with ECM remodeling capability, but a different morphology that the classical F-actin dots. These structures present a linear organization and are associated with collagen I fibrils. They are also composed of scaffold elements of invadosomes (TKS5, SRC, WASP, ARP2/3, cortactin) and metalloproteinases (MT1-MMP, MMP2). Linear invadopodia depend on CDC42 activation and DDR1 [47], but they lack many other elements like vinculin, paxilin, or integrins [37].

### 3.1. Podosomes and Their Related Functions

Podosomes are cellular actin structures characteristic of non-transformed cells, including osteoclasts [64,65,66,67], macrophages [68,69], megakaryocytes [70], and dendritic cells (DCs) [71]. The term podosome was coined to describe actin structures in the ventral cell surface of Rous Sarcoma Virus transformed fibroblasts with the SRC oncogene [72]. Later, these structures were also observed in many other cell types under natural conditions [73]. Advanced imaging technologies have allowed the capture of in vivo images of invadosome structures (TKS5, cortactin, and actin-positive structures) during intestine organogenesis in zebra fish models [35,74] and *Caenorhabditis elegans* gonad organization [35]. Podosome formation has been also linked to other important normal processes such as synapsis formation, neural cone elongation, antigen recognition, and cell fusion. Podosomes can be induced or spontaneously formed. In cells of myeloid lineage, for example, the mere adhesion to a substrate is enough to trigger podosome formation. Integrins would mediate cell adhesion to the ECM and activate outside-in signaling pathways (GTPases and kinases) to induce podosome formation directly (Figure 1, 1 and 2) [75]. Other non-hematopoietic cells can also form podosomes in response to extracellular cues such as growth factors, matrix mechanical properties, or other stimuli [35,37,76,77,78].

Loss of podosome formation in hematopoietic linages is associated with serious diseases, e.g., WASP mutation is the origin of Wiskott–Aldrich syndrome (WAS) [79]. In zebra fish embryos, TKS5 morphan mutants present many developmental defects (e.g., head, eye cardiac pigmentation) [80]. Frank–ter Haar syndrome (FTHS) is an autosomal recessive disease linked to TKS4 abnormalities and podosome formation during embryonic development [1]. In addition, organogenesis defects were observed in neural crest cells (NCC) in TKS4 and TKS5 knockouts [81].

The ultrastructural composition of podosomes is very complex, with different domains, regions, and proteins (Figure 1). Podosomes present a special structure, called podosome caps, which are formed by formins (INF2), formin related proteins (FMNL1), and supervillin [64,76]. In macrophages, this structure regulates podosome growth, degradation, and contractile forces, acting as a vesicle reception center [68,69,76]. Other formins, mDIA2 or FBP17, are also implicated in actin elongation machinery recruitment (WASP-WIP) and microtubule dynamics [70].

In macrophages, supervillin and myosin IIA determine the presence of two different podosome subpopulations: the precursors, which are larger and located at both the periphery and the leading edge, and the successors, derived from the precursors and located toward the center of the cell [68]. In addition, lymphocyte-specific protein 1 (LSP1) modulates adhesion, migration, and podosome turnover in primary macrophages by the regulation of actomyosin contractility [69].

In order to expand blood vessels during neo-angiogenesis, endothelial tip cells overexpress VEGF receptors and down-regulate Notch signals to form podosomes [76,77]. It has been recently proposed that podosome rosettes in tip cells degrade collagen IV basement membrane during the sprouting process of breaching the basement membrane, and then during anastomosis [76].

Osteoclasts are responsible for bone resorption by attaching to the bone surface while moving through it [66]. In the first step of adhesion, osteoclasts form podosome aggregates that evolve into rosettes. Later on, these structures are fused to form a stable, degrading structure over mineral matrices known as sealing zones [82]. This process results in the generation of a membrane-rich ruffle border, surrounded by a sealing zone composed of podosomes organized in actin rings [64,65,66,67]. This podosome distribution is critical for bone resorption, while ECM binding to integrins and CD44 allows osteoclast migration through the bone surface [64]. Actin filaments are stabilized by crosslinker proteins such as α-actinin, vinculin, paxillin, or phosphorylated L-plastinin, forming concentric rings in contact with integrins, in structures known as adhesion plaques [64,83]. These plaques are also stabilized by myosin II and α-actinin in co-axial segments, which form the podosome cloud in the peripheral region of podosomes [64]. Myosin II, MLKC, and supervillin also participate in podosome stabilization, but they can induce podosome dissolution depending on signaling and traction forces [68]. Several guanine-nucleotide exchange factors (GEFs) stimulate podosome organization in rosettes, and modulate membrane trafficking or cytoskeleton modifications through cortactin signaling [70]. Other GEFs bind to β3-integrins and modulate osteoclast differentiation [70,84,85]. Recently, it has been demonstrated that β-PIX, a RAC1/CDC42 activator, negatively regulates FA and promote lamellipodium development and invadopodia [85,86].

During first steps of adhesion, fibroblasts form integrin clusters, where F-actin cores start to precede the formation of podosome rings [51]. In the absence of traction forces, Arg-Gly-Asp (RGD)-activated-integrins clusters lead to local enrichment of PI3K regulatory subunit p85β and production of PIP3. This negatively charged domain induces the activation of N-WASP to promote actin polymerization in a formin-independent manner by ARP2/3. p85β is recruited by auto-phosphorylation FAK/PYK2, while SRC activation is not necessary [51]. Traction force generation inhibits podosome formation and transforms them to FA structures as result of RHOA-GTP activation. ARAP3, a RHOA-GAP, binds PIP3, and is located in podosomes but not in FA under free force conditions. Thus, PIP3-ARAP3 binding in podosomes would inactivate RHOA to stabilize podosomes [51].

### 3.2. Integrin in Podosome

Integrins are proteins present in classical invadosomes, which form the adhesion ring in the upper domain of the structure (β2 and β3) and in the podosome core (β1) [35,87]. Integrins, and their related proteins’ clustering, induce signaling pathways to control cell adhesion, migration, and invasion. Integrins and CD44 are the main transmembrane adhesion receptors found in podosomes (Figure 1) [35,67,76]. The presence and function of integrins and their regulatory proteins in podosome formation have been massively described [3,36,39,75,87,88,89,90,91,92,93,94,95]. Proteomic analysis shows that CD44 and β2-integrin are the adhesion receptors detected in macrophagic podosomes, together with many other invadosome molecular compounds such as Arp2/3, cofilin, vinculin, zyxin, talin-1, kindlin-3, and myosin IIA [96]. In contrast, no integrins were detected or enriched in murine fibroblast podosomes, although some integrin binding partners such as talin and vimentin were detected [97].

Integrin expression in podosomes depends on both the type of matrix and the cell type. β1-integrin is highly expressed in megakaryocytes and it binds to several collagen isoforms, fibronectin, and laminin. β2-integrin mediates the formation of podosomes in macrophages, binding to fibrinogen, and β3-integrin binds to osteopontin, vitronectin, and fibrinogen, preferentially in osteoclasts [70,76]. Interestingly, in order to block podosome formation in osteoclasts, all three integrins (β1, β2 and β3) must be simultaneously silenced [95]. Specific integrin expression, mainly of β2-integrins, has been also associated with podosome-like structures on mesodermal progenitor cells associated with degradation areas, and MMP9 in a FAK/ERK1/2 signaling-dependent manner [98]. In all cases, binding between integrins and their ligands activates several phosphorylation cascades and mediates almost every step of podosome formation and maturation [70]. Linear invadosomes are degradative structures present in endothelial cells and embryonic fibroblast cells, associated with collagen fibers. It is noteworthy that β1 and β3-integrins are present in focal adhesion, but not in linear invadosomes. Furthermore, integrin blockade, or silencing, does not alter the linear invadosome formation [37]. ILK is essential for integrin clustering and adhesion ring formation, but not for actin core formation of immature DCs (iDCs) podosomes. ILK silencing negatively regulates PI3K recruitment and subsequently WASP activity in podosomes [99].

#### 3.2.1. β1-Integrin in Podosomes

This integrin plays a key role in the assembly of podosome rosettes. Mouse embryonic fibroblasts with constitutively activated SRC form invadosomes associated with β1-integrin expression and PKC regulation [100]. High α6β1 integrin levels enhance podosome lifespans during neoangiogenesis [101], while in quiescent vessels the expression is lower [102]. In response to VEGF, endothelial cells increase α6β1 and degrade the basement membrane of Collagen-IV to create space for new endothelial cells [77,101]. Many studies have demonstrated that podosome blockade has severe effects on all these processes [101,103].

Eosinophils are immune cells associated with allergic responses. These α4β1 (also known as VLA4) integrin-expressing cells form podosome-like structures over endothelial cells in a process dependent on endothelial VCAM1 expression. Unlike adherent fibroblasts, eosinophil podosomes can degrade VCAM-1 from endothelial cells in an ADAM8 metalloproteinase-dependent manner. Eosinophil α4β1 integrin is located in podosome structures, while α4β7 or αDβ2, other VCAM1 partners, present a diffuse distribution [104]. In culture, human macrophages can form podosomes in 2D and 3D collagen environments, but the displayed structures are different. In a 2D environment, they spontaneously form big rosettes or individual podosomes, but when they are studied in a 3D environment, they form big individual podosomes [105,106]. Literature supports that β1-integrin and CD44 are the main collagen receptors, while β2-integrin is only occasionally found in individual podosomes formed in 2D environments. Interestingly, in a 2D environment, β1-integrin and CD44 localize, surrounding podosome rosettes, while in a 3D environment, both proteins are at the tip of the podosomes [106]. In primary megakaryocytes, α2β1 ligand binding promotes podosome formation, while WASP-deficient megakaryocytes present defects in proplatelet formation in collagen-I environments [94].

#### 3.2.2. β2-Integrin in Podosomes

Members of the β2-integrin family include αLβ2 (CD11a/CD18, LFA-1), αMβ2 (CD11b/CD18, Mac-1, CD3), αxβ2 (CD11c/CD18, p150, 95, CR4) and αDβ2 (CD11d/CD18). They are expressed in most white blood cells (WBC), as well as in smooth muscle cells, and have a crucial role in podosome formation [88,91]. For instance, vinculin and β2-integrin are recruited to the surrounding area of the podosome actin core in response to ICAM-1 (ligand of β2-integrins) and fibronectin (ligand of β1 and β2-integrins) binding [88]. In human monocyte-derived macrophages and monocyte-derived DCs, αMβ2 and αXβ2 bind fibrinogen, and they are found in the adhesion ring of podosomes [107]. It has been described that podosome formation and β2-integrin clustering fail in macrophages, polymorphonuclear cells, and DCs of WAS patients [91], suggesting a critical role of WASP in the recruitment of integrins to form mature podosomes [90,91].

Endothelial barriers are critical walls that immune cells must cross. During transcellular diapedesis, leukocytes, lymphocytes, monocytes, and basophils insert their podosomes into endothelial cells to choose the most suitable region to cross. β2-integrin and its partners in endothelial cells are vital in this process, because inhibition of ICAM-1, PECAM, or some podosome regulatory proteins reduces or cancels cell diapedesis [108,109]. Matrix stiffness is also a factor that regulates podosome formation through β2-integrin, but not β1, expression in iDCs [110]. Recruitment of αMβ2 to iDCs podosomes is a critical step, because a solid adhesion to endothelium is necessary to access secondary lymphoid organs. During maturation of DCs, the cysteine protease cathepsin X moves to the plasma membrane and associates with αMβ2, allowing the activation of this integrin receptor [93]. Inhibition of cathepsin X prevents adhesion and provokes podosome disassembly [90,93]. Moreover, IL-5 stimulates eosinophil adhesion to periostin through αMβ2, which is up-regulated by lymphocyte Th2 cells to form podosomes [111]. Furthermore, podosomes have been also found in mesodermal progenitor cells (MPCs), the precursor cells of mesenchymal stromal cells, and specifically expressed αLβ2, αMβ2 and αXβ2 to adhere themselves to the epithelium [98].

#### 3.2.3. β3-Integrin in Podosomes

β3-integrin is vital for embryonic development, bone remodelling, platelet function, and angiogenesis. For instance, the interaction of β3-integrin and kindlin-2 to promote podosome formation in endothelial cells has been demonstrated in vitro using optogenetic models [92]. Mice deficient for the FA protein kindlin present αv, β1 and β3-integrin subunit reduction, and develop an important deregulation in osteoclasts’ podosomes and bone resorption [64]. In osteoclasts, β3-integrin mediates cell adhesion and activation of growth factor signaling pathways that stimulate the activation of CDC42, RHO and RAC, among other small GTPases. Mice lacking β3-integrin develop osteoporosis as result of defects in bone resorption [66], although β3^−^/^−^ mouse embryonic fibroblasts still form small podosomes in constitutively active SRC conditions [100]. αvβ3 is involved in recognition of the ECM, in the maintenance of the sealing zone, and in the organization of the cytoskeleton [65,112]. Sealing rings are integrin-rich, subcellular structures associated with bone remodeling by osteoclasts, and integrins (mainly αVβ3) are essential components of the structure [83]. Although αvβ3 is the main integrin found in osteoclasts, its absence does not disrupt bone resorption, suggesting that other integrins may have overlapping functions [67]. In fact, it has been demonstrated that αv, β1, and β2 play also crucial roles in bone resorption, and their individual ablation results in an important reduction of the resorptive activity [95]. αvβ3 is found inactive in podosomes during ECM attachment, and is activated and re-colocalized in lamellipodia when it binds its ligand [65]. Nevertheless, other authors postulate that actin ring formation depends on the receptor CD44, as well as ARP2/3 and cortactin. αvβ3 would be located in the adhesion ring, co-localizing with paxilin and vinculin, while CD44 would be in the tip of the podosome [35,64,67]. This distribution has been associated with podosome belt structure in mature osteoclasts over non-mineralized substrates [45]. αII-Spectrin is a component of the invadosome cloud around the actin core of microvascular endothelial cells, and silencing it increases adhesion ring dynamics. αII-Spectrin depletion reduces the immobilized β3 location in adhesion rings, increases the formation of unstable podosomes, and reduces ECM degradation [113].

It has been suggested that the recruitment of β3-integrin, paxilin, and cortactin represents the first step in the assembly of podosomes, and is followed by the recruitment of F-actin in the core and α-actinin, and the increase of β3-integrin in the podosome cloud [64,114]. Phosphorylation of several residues in the β3 cytoplasmic domain of αvβ3, as result of ligand-binding, induces the recruitment of PYK2 (a FAK family member) to podosomes. PYK2 phosphorylation allows SRC binding and podosome assembly [65]. Other tyrosine kinases, such as SYK, a member of ITAM family, also participate to transduce the signal to RAC [115]. αvβ3 has also been shown in complex with VASP (vasodilator stimulated protein), which is a substrate of PKG1 (cGMP-dependent protein kinase 1), which, in turn, is stimulated by nitric oxide, considered the main bone turnover regulator [116]. PIP2 and PIP3 levels, produced in response to αvβ3 signaling by SRC and RHO-GTPases, modulate gelosin and WASP, and, in consequence, the actin ring formation or the podosome assembly/disassembly for osteoclast migration [117]. αvβ3, VAV3 (RAC-GEF) and SYK kinase complex, as result of SRC activation, modulate actin cytoskeleton by RAC1 action [54].

#### 3.2.4. β4-Integrin in Podosomes

Epithelial cells interact with ECM by focal contacts and hemidesmosomes, which contain podosome-like structures. For instance, squamous and transitional epithelial cells have hemidesmosomes with α6β4 integrin in order to bind laminin-5. These podosome-like structures are also present in the actin core, with α3β1 integrin around it. During the assembly of hemidesmosomes, β4-integrin is located at the base of the actin core, but when cells are induced to migrate it becomes co-localized with dynamic actin at the leading edges [118].

#### 3.2.5. α-Integrin in Podosomes

Only the α3-integrin subunit has been described individually as a podosome element. It was described in the adhesion ring of podosomes of an oral SCC cell line derived from a primary gingival tumor. It presented in the same location as β1-integrin, and it was proposed that α3β1 is a functional heterodimerization found in the podosomes [119].

### 3.3. Invadopodia and Their Related Functions

Invadopodia are the bad members of the invadosome gang. They are actin-rich tumoral structures with ECM degradative ability associated with metastases and malignant tumors [1,39]. These structures participate in ECM degradation and the extra- and intravasation processes of tumor cells. Many authors postulate that invadopodia are one of the first steps of tumor dissemination [39,120]. Primary tumor cells isolated from patients have demonstrated the formation of invadopodium-like structures with the classical markers of the invadosome [89,121,122]. Recently, a strong correlation between invadopodia and a metastatic signature expression was found, suggesting that invadopodium-related genes could potentially be used as prognostic markers or new potential anti-metastatic targets [121]. Integrin and MMPs elevated expression correlates with recurrence and poor prognosis in breast cancer [123]. Pharmacological inhibition of different targets in invadopodium structure has demonstrated good results against metastases in mouse models of lung cancer, bladder, melanoma, fibrosarcoma, pancreas, and breast cancer [124,125,126,127,128] with a reduction of circulating tumor cells as result of inhibition of tumor intra- [129] and extravasation [124].

### 3.4. Integrins in Invadopodia

Invadopodia, like podosomes, are spontaneously formed by tumor cells or as a result of different stimuli: soluble growth factors, cell–cell contact or ECM properties, and signaling. Structurally, invadopodia are described as actin structures in the ventral surface of cells, where they contact with the ECM. They have a central core of polymerized branched actin with many regulatory proteins around the structure, and MMPs for ECM degradation [3,89]. Integrin or adhesion receptor activation induces signaling cascades though linked proteins (actinin, vinculin, and talin) to the FAK/SRC axis (Figure 1) [3,89]. The role of FAK in invadopodium formation is a bit controversial, because many authors postulate negative effects, while others propose an inductive function [3,49,130]. FAK controls SRC phosphorylation without being located in invadopodia. PYK2 is a FAK homologue that controls podosome dynamics and stabilization [131]. Recently, it has been demonstrated that PYK2 induces invadopodium formation. It controls cortactin phosphorylation in a SRC/ARG-dependent manner in breast cancer cell line MDA-MB-231 and other tumor cells [132]. SRC, and its phosphorylation status, RHO-GTPases (RAC, RHO, CDC42…), PI3K, ERK and/or PKC transduce signals that converge in cortactin, a classic invadopodium marker. Cortactin modulates actin polymerization and branching mechanisms (N-WASP, WIP, ARP2/3, calpain, cofilin, etc.) (Figure 1) [36,120,133,134], and promotes ECM degradation by MMP2, MMP9 and MP1-MMT, but also ADAMs, serine proteases, or cathepsin cysteine proteases [58].

The first description of integrins and their regulatory proteins in invadopodia was reported twenty years ago [135]. The role of integrins in invadopodia of tumor cells is multifold, and it is postulated that it depends on the matrix, stimulus, and/or cell types. Integrins usually form an adhesion ring podosome-like structure [136]. However, integrins can localize to invadopodium cores, depending on the cell and matrix types used [35,136]. Integrin recruitment to invadopodia usually occurs after the initiation stages and before matrix degradation [36,39]. General consensus postulates that the adhesion ring can contain different integrins, such as α2, α3, α5, α6 and β1, but not β3 [49,136], although some studies also report the implication of β3-integrin in invadopodium formation [137,138]. β5-integrin has been also described in the invadopodium “head” of a patient-derived oral SCC cell line which cells are undergoing epithelial–mesenchymal transition. In invadopodia, β5-integrin would interact with an αV subunit to form a functional heterodimer [119]. αV-integrin was found to be elevated in proteomic analysis of prostate resistant cancer cell lines, and its blockade inhibits cell invasion [139]. β1-integrin was also detected in GBM vesicles associated with invadosome proteins such as ACT3 [140]. Heterodimer αVβ1 was proposed to be an invadopodium base, forming a complex with ARF6, IQGAP, ILK, and filamin A [141].

Integrin ligand binding generates outside-in and inside-out signals which cooperate to regulate cellular process [25,142,143], and an intense protein recruitment allows formation of membrane microdomains to promote invasion and invadopodium formation [60,144,145,146,147,148,149,150]. It has recently been demonstrated that invadopodia are chemosensing structures that modulate tumor cell extravasation in chorioallantoic membrane model invadopodia formation with intravital microscopy [151]. Authors observe that PAK1 activation regulates cofilin and myosin light chain (MLC) to control invadopodium disassembly, but it also modulates the chemotactic response to EGF and GABA to promote breast tumoral traffic through the membrane [151]. It has been demonstrated that integrins also regulate PAK1, cofilin, and MLC [146,152,153,154], and cooperate in cells’ invasion with growth factors [155]. Exosomes are cell–cell communication factors necessary for invadopodium formation and MT1-MMP secretion [156], Moreover, TKS5–invadopodium inhibition has been demonstrated to reduce exosome release [157]. Recent published works demonstrate that exosomes, secreted by tumor cells, fuse with resident cells to prepare the niche for tumor metastasis [158,159]. Proteomic analysis of these exosomes showed that they contained integrins, and their pattern was associated with organ-specific distribution of metastasis. While liver metastasis was associated with exosome αvβ5 integrin, lung metastasis was linked to α6β4 and α6β1. Exosome-integrin reduced uptake decreased metastasis and prometastatic signal pathways [158]. Furthermore, it has also been described that exosomes secreted by cancer stem cells (CSCs) or cancer-associated fibroblasts (CAFs) are more relevant for tumoral invasion that exosomes from the tumor [160]. All data demonstrate that integrins, stromal cells, the microenvironment, and invadopodia form a feedback loop to control and/or promote cancer metastasis.

Integrin expression and location are altered in many diseases, therefore, therapies against these proteins have been designed and are in advanced phases of development [22]. Integrin expression varies between tumors, with malignant tumors usually presenting overexpressed integrin patterns [161]. In particular, melanoma and glioblastoma express elevated amounts of αvβ3, colorectal carcinomas overexpress αvβ6, αvβ5 is overexpressed in breast, lung, and melanoma, while αvβ8 frequently appears in brain metastasis [21,161]. Anti-integrin therapies have been developed and tested with different results. Cilengitide is an antagonist of αvβ3/β5 integrins, which failed to demonstrate clinical efficacy in a phase III glioblastoma trial. Moreover, Etaracizumab, also known as Abegrin, is another anti-αVβ3 integrin that did not demonstrate better results over dacarbazine in a phase II trial against stage IV metastatic melanoma [162]. Volociximab, an antibody against α5β1 integrin, obtained good results in an ovarian and peritoneal cancer phase II trial, and it is postulated that it could be useful in treating glioblastoma multiforme (GBM) [22,163]. Intetumumab (CNTO95), an anti-αv molecule, inhibits angiogenesis in solid tumors [164], and PF-046055412 is another safe anti-α5β1 tested on non-hematologic solid tumors [22,165]. Finally, ATN-161 is a five amino acid peptide (Ac-PHSCN-NH_2_) derived from fibronectin that inhibits α5β1integrin signaling, and had good toxicity results in a phase I trial against solid tumors [166].

#### 3.4.1. β1-Integrin in Invadopodia

The β1 subunit is the integrin most often reported to be associated with invadopodium formation in tumors, and of which blockage or silencing reduces invadopodium formation in multiple cancer types [49,60,100,136,154]. α2β1, α3β1, α5β1 and α6β1 integrins are overexpressed in glioblastoma tumors, and anti-β1 antibodies reduce tumor invasion [89]. In melanoma cells cultured in vitro, α3β1 interacts with seprase, also known as fibroblast activation protein (FAP), in invadopodia [135]. Furthermore, it is located around the actin core of the podosome-like structures of 804G carcinoma cell [118]. Fibronectin binding to α5β1 induces invadopodium adhesion structure or degradative activity, depending on melanoma cell line [135,167]. This integrin was observed in adhesion ring of breast cancer cell lines, and in the invadopodia puncta when cells present activated SRC [136]. α6β1 integrin is a laminin partner that transduces the signaling necessary for invadopodium activity in LOX melanoma human cells by inducing the translocation of β1-integrin, gelatinase, and seprase to the plasma membrane [168]. The CAIX protein (carbonic anhydrase IX) is a hypoxia-induced factor of which the function is to regulate intracellular pH. It has been demonstrated that CAIX interacts with β1-integrin and MMP14, and is colocalized with cortactin, TKS5, and MMP14 in invadopodia [169]. β1-integrin silencing decreases the capability of tumor cells to form invadopodia after C16-laminin peptide exposition [170]. These data were confirmed by Artym and coworkers, who observed that invadopodium formation induced by high-density fibrillar collagen was reduced when β1 and α2-integrin were blocked with antibodies in breast cancer cells [171]. However, when cells were grown over gelatin layers, the main integrins implicated in invadopodium formation were β1 and α5 integrins [171].

β1-integrin performs functions at different stages during invadopodium formation.

A. It is associated with ARG phosphorylation of cortactin by direct protein–protein interaction [39,49,53]. Combined β1-integrin and EGFR interaction/signaling is necessary for ARG activation in breast cancer cells. The β1-integrin cytoplasmic tail blocks Y272 ARG autophosphorylation, which allows its phosphorylation in Y466 by SRC to promote invadopodium formation [154].

B. This integrin is also important in NHE1/moesin recruitment to the invadopodium core [39]. An increased level of Erzin p(T567) was detected in aggressive human tumors and invadopodia as a result of β1-integrin activation. Erzin interacts with NHE1, β1-integrin, EGFR and NHERF1 to form a complex in invadopodia associated with lipid raft formation [60].

C. β1-integrin also regulates SRC activation by indirectly kidnapping FAK at focal adhesion points. MENA^INV^ and α5β1 modulate FAK/SRC interaction and their signaling transduction pathways [39].

D. It has been demonstrated that β1-integrin promotes metalloproteinase secretion at invadopodia [136]. This integrin, in association with ILK, IQ-domain GTPase-activating protein 1 (IQGAP1), and the formin mDIA1, modulates the vesicle traffic and metalloproteinase release at invadopodia, mainly MT1-MMP [36,49,136]. ILK silencing or RGD-inhibitor peptides reduce IQGAP recruitment to the invadopodium core [136]. CD44 cooperates with α5β1 integrin to promote invadopodium formation over fibronectin [39]. CD44 isoform is also associated with cortactin phosphorylation and MT1-MMP recruitment to invadopodia, and it also binds MMP9 in lymphoma cells [39]. Seprase and other collagenases interact with β1-integrin to promote matrix degradation [53]. β1-integrin inhibits MT1-MMP endocytosis in human endothelial cells and induces the Rab8-mediated exocytosis mechanism. Furthermore, it is proposed that β1-integrin could anchor MMPs in the plasma membrane, in cooperation with CIP4 [49]. A strong positive correlation was observed between degradation area and adhesion ring formation, mainly β1-integrin, in breast cancer cell lines [136].

E. β1-integrin also participates in mechanosignaling. Cofilin inactivation and β1-integrin over-expression were detected under mechanical stimuli in the HT1080 human fibrosarcoma cell line [153]. Kindlin 2 directly binds β1-integrin to regulate its bidirectional signaling over dense collagen matrix [171]. Migfilin interacts with phospho-kindlin 2 and β1-integrin to release Filamin A from the cytoplasmic tail of integrin to activate its signalling. Migfilin and phospho-kindlind 2 are located in the invadosome, where they are fundamental elements in integrin-mediated signalling [171].

#### 3.4.2. β3-Integrin in Invadopodia

The role of β3-integrin in invadopodium formation is very controversial, although many cancers show dysregulation of αVβ3 integrin [26,161]. Some authors have proposed that this integrin is not implicated in invadopodium development, while other authors have observed an important role in same tumors. In melanoma cell line A375M, αVβ3 is associated with increased invasion and collagenase secretion [172]. β3-integrin, but not β1, was observed in adhesion rings around the F-actin core of the osteoclast isolated from surgically excised osteosarcoma giant cell bone tumor [73]. β3-integrin is mainly located in the adhesion ring, although it has also been detected at the tips of the invadopodia [138].

β3-integrin’s functions during invadopodium formation are also, like β1, associated with different stages.

A. αVβ3 integrin interacts with MT1-MMP to promote MMP2 activation in breast cancer cell lines [173], but it also interacts with MMP2 to form a complex in blood vessels of melanoma tumors in vivo [174].

B. In breast tumor cell lines, collagen IV activates Dishevelled-associated activator of morphogenesis 1 (DAAM1) and RHOA to promote haptotaxis. DAAM1 is a formin family member often localized to invadopodia of breast cancer cell lines, where it binds αVβ3 to promote invadopodium formation though the modulation of actin polymerization and free barbed end formation [26]. The αVβ3 inhibitor peptide (Cyclo-RGDfK) is also efficient to block invadopodium formation and haptotaxis of breast tumor cell lines over collagen IV [26].

C. SNAIL2 is a transcription factor in which silencing is associated with inhibition of invadopodium formation. SNAIL2 is also down-regulated after αVβ3 or fibronectin silencing. Chloride intracellular channel (CLIC1), which is up-regulated in many tumors, interacts with αVβ3 and regulates fibronectin-αVβ3 interactions and fibronectin assembling to promote metastasis [163,175]. Both proteins are located in the adhesion ring of invadopodia in fibrosarcoma and kidney cancer cell lines, as well as renal tumor cells isolated from two patients [122,163]. In these cells, integrin silencing reduces the formation of invadopodia and matrix degradation [122,137]. In this context, fibronectin and αVβ3 would regulate MLCK and RHOA signals to control vesicle transport, actin polymerization, and transcription factors associated with EMT to form invadopodium structures and promote cancer metastasis.

D. αVβ3 over-expression correlates with invadopodium formation in renal carcinoma, sarcoma, and glioblastoma cell lines. αVβ3 siRNA reduces invadopodium formation and cell proliferation in these cell lines, and these effects were not detected in β1-silenced cells [137]. β3-integrin is up-regulated after TGF-β treatment in lung cancer cells [145]. Invadopodium formation and FAK, ERK, SRC and cortactin signaling are also up-regulated in these cells cultured in 2D and 3D environments [138]. β3-integrin antibody blockade, but not β1, abrogates invadopodium formation in lung cancer cells. This effect was also observed after integrin silencing with a shRNA, while integrin re-expression restored invadopodium formation [138].

#### 3.4.3. β8-Integrin in Invadopodia

β8-integrin subunit heterodimerizes exclusively with αV-integrin, and it binds to latent TGF-β. αVβ8 expression is altered in malignant brain tumors and metastases [176]. β8-integrin interacts with the actin scaffolding proteins spinophilin (Spn), for which silencing induces invadopodium formation in glioblastoma cells and alters RAC1 signaling [176]. The β8-integrin cytoplasmic tail has lost the capacity of inside-out signaling, so, after ECM binding, it remains constitutively activated [177]. It is postulated that β8-integrin localizes Spn in the leading edge to promote invadopodium disassembly and reduce glioblastoma invasion metastases [176].

#### 3.4.4. α-Integrins Subunits in Invadopodia

α3- and αV-integrins have been described individually as invadopodium elements in an oral squamous carcinoma cell line derived from a recurrent gingival tumor. While α3 appeared surrounding the invadopodium membrane, αV was located at the “head” of the invadopodia [119]. GMB are diffuse and infiltrative tumors in which cell lines models present a strong degradative capacity. α5-integrin was overexpressed in the plasma membrane of highly metastatic cell lines, and ITGA5 siRNA silencing reduced the invadopodium and degradative activity after EGF treatments [163]. Transcription factors, such as Ets-2 and Ap2, which control α5 expression, are also up-regulated in these GBM-derived cell lines [163]. Both α5-integrin and the β subunits interact with nischarin [149]. Nischarin is a cytosolic protein with tumor suppressor properties, for which mutations are associated with decreased patient survival [178]. In breast cancer cells, nischarin reduces invadopodium formation when it is over-expressed, but it also increases their formation when it is silenced. This protein modifies invadopodia at different levels, and alters metalloproteinases (MMP1, 2 and 9), structural elements (TKS4 and AFAP-110), or secreted factors and fibers in a cell environment [149]. Interestingly, nischarin silencing increases α1, α4, α5 and α7 integrin expression, but reduces α2 [149].

## 4. Microenvironment Factors Associated with Invadosome Formation

ECM and cells present in the surrounding tumor stroma contribute to cancer progression. Non-tumoral cells (fibroblasts, macrophages, endothelial cells) and structural aspects of ECM (composition, density, cross-linking, rigidity and stiffness) are factors that have been implicated in tumor malignancy and migration by mechano-transduction signals or ECM remodeling [50,179]. These factors modulate cell activities such as migration or invasion, and, subsequently, associated cell structures as invadosomes.

### 4.1. ECM Compounds

Collagens, laminins, fibronectin, hyaluronic acid, entactin/nidogen or heparin sulfate proteoglycans are extracellular matrix compounds, the interactions of which with the integrin heterodimers modulate their conformational configuration and functionality [180]. These molecules potentially impact the progression, invasion, or metastasis of cancer [181]. One integrin can bind different ligands, and the same ligand can interact with different integrins [17]. Integrins recognize specific sequences in their ligands and, on this basis, anti-integrin therapies have been developed. The inhibitory peptide Cyclo-RGDfK interacts with αVβ3, and it has been demonstrated to be an efficient blocker of invadopodium formation in breast cancer [26]. This has also been observed with other inhibitory peptides (cRGD) for β1-integrin in linear invadosome formation [37,136].

#### 4.1.1. Collagens

These molecules are the main components of ECM and basement membrane. They are ligands for some integrins, and have been demonstrated to regulate invadopodium formation. For instance, megakaryocytes increase podosome formation in collagen matrices, in addition to other matrices such as fibrinogen [70]. Liu observed that collagen-I, a β1-integrin ligand, inhibits invadopodium formation in rat bladder carcinoma cells [182]. Nevertheless, other authors have observed that fibrils of collagen I can promote linear invadosome formation in an integrin-independent manner in many cellular types, including tumor cells, and the induction is faster than using soluble agents [37]. High-density fibrillar collagen deposits have been observed in stroma adjacent to tumors [183,184], and these dense deposits have also been demonstrated to induce invadosome formation in an integrin-dependent manner in breast tumor cells and primary human fibroblast [171]. Linear invadopodia depend on collagen I [37], and only the Discoidin domain receptor 1 (DDR1) is responsible for the interaction between the collagen and the formation of these linear structures [47]. Bladder tumor cells secrete collagen IV alpha 1 (COL4A1) and collagen XIII alpha 1 (COL13A1), which are α1β1 and α2β1 ligands. Knock down of these collagens has been demonstrated to be associated with decreased invadopodium formation, reduced matrix degradation, and decreased tumor invasion [185]. However, other authors have observed increased invadopodium formation in response to collagen type IV in breast cancer cells associated with increased RHOA activity and ADDM1-αVβ3 interaction [26]. Recently, it has also been observed that collagen IV induces SRC-dependent podosome formation in microvascular endothelial cells with matrix proteolytic activity upon VEGF-A exposure [103]. Other collagens may be implicated in these processes, as, for instance, collagen VIII binding to β1-integrin receptors suppresses RHOA signaling, resulting in stimulation of MMP-2-dependent smooth muscle cell migration [186].

#### 4.1.2. Fibrin and Fibrinogen

Both fibrin and fibrinogen molecules are β2- and β3-integrin ligands associated with podosome formation in macrophages [187] and other cell types, and also promote tumor invasion, metastasis, and angiogenesis [122]. Fibronectin, which is secreted to form fibrils and a suitable cell environment, is another binding element of β1- and β3-integrins. Fibrin embedded tumor cells presents high levels of αVβ3 and fibronectin expression. Moreover, fibrin-fibronectin-αVβ3 complex controls invadopodium formation and induces signal cues for cell proliferation and metastasis gene expression [137,188].

#### 4.1.3. Laminins

Laminins are basement membrane glycoproteins that interact with different integrins, such as αVβ3, α5β1, α3β1, α6β1, α7β1, α6β4 and α2β1 [189]. It has been reported that AG73 and C16 laminin-111-derived peptides induce invadopodium formation in adenoid cystic carcinoma (CAC2), fibrosarcoma, and squamous carcinoma cells [170,190]. C16 peptide interacts with β1-integrin and induces RAC, SRC, and ERK1/2 signaling, increasing cortactin phosphorylation [170]. These data are in concordance with the invadopodium signal pathways activated by integrins in lung cancer cell lines [138]. In addition, β1-integrin siRNA silencing reduces invadopodium formation in CAC2 cells after C16 treatment [190]. Furthermore, laminin-322 also modulates invadopodium formation in 804G rat bladder carcinoma cells [182]. This laminin is an autocrine secreted molecule during epithelial remodeling, and it is also associated with cancer progression [191,192]. β1-integrin and their ligands negatively regulate invadopodium formation in rat bladder carcinoma cells, because invadopodium inhibition was observed when β1 integrin, but not α6, αv or β4 integrin, was blocked. This effect was accompanied by a reduction of SRC activation and FAK up-regulation [182]. These data support the hypothesis that integrin–ligand binding and FA formation negatively regulate invadopodium generation by FAK and SRC signaling, and the reduction of laminin-322 secretion would reduce invadopodium-mediated invasion [182].

### 4.2. ECM Properties

#### 4.2.1. ECM Rigidity

This matrix factor has been demonstrated to be associated with development of invasive carcinomas in human and mouse models of breast cancer [193,194,195,196]. Stroma surrounding tumor is often more rigid and dense, with increased collagen and fibronectin presence [197]. Actomyosin contractility is increased by rigidity, a process mediated by ROCK phosphorylation of MLCK and NMII [193]. These proteins, together with traction forces, mediate ECM degradation by invadopodia [130,198]. Moreover, it has been also described that matrix stiffening and collagen crosslinking are associated with focal adhesion and integrin signaling to promote cell invasion [199]. Invadopodium formation was detected in rigid, but not soft, substrates when cells overexpressed FAK and p130CAS [130]. This is in concordance with increased invadopodium formation, and degraded areas, when breast cancer cells were seeded over denser and more rigid matrixes of gelatin and fibronectin [130,179]. It is postulated that greater density would increase the integrin binding sites, generating signals to promote invadopodia [200]. However, increasing rigidity without modifying fiber number still increases invadopodium formation [130]. Furthermore, it is hypothesized that the central actin core exerts pushing forces to the matrix, which are also associated with actomyosin contractility around the actin core, and this effect is associated with adhesion rings [196]. Cells use mechanosignals to evaluate their surrounding matrix and can activate the myosin II-FAK/CAS pathway to increase invadopodium formation in a rigidity-dependent manner [130]. Podosome ROCK1 is involved in traction force signaling and modulates actomyosin contractility and rigidity inducing invadopodium formation. ROCK2 also participates, but in a different way. It mediates pLINK signaling to modulate invadopodium maturation by MMPs regulation and matrix degradation [193]. It is postulated that forces and matrix stresses are transferred to cells, in an outside-in signaling pathway, by integrins in the adhesion ring of the podosome [201], although zyxin, paxillin, α-actinin, or vinculin are also candidates to participate. Podosomes also transfer forces to the matrix through ROCK-NMII [193] inside-out signaling. However, it has been also observed that podosomes can be formed in the absence of traction forces when integrins bind to RGD peptides in liquid media. Thus, integrin clustering induces FAK/PYK2 autophosphorylation, p85 recruitment, and RHOA inhibition, leading to actin polymerization [51].

#### 4.2.2. Fiber Crosslinking

Matrix crosslinking is a factor related to matrix rigidity that negatively regulates invadopodium formation, penetration, and matrix degradation [202]. Altogether, cross-linked matrix and basement membranes are effective barriers for invadosomes [202,203]. In vivo cross-linkers such as lysil oxidase (LOX) or transglutaminases are secreted to the ECM by tumor stromal cells, e.g., macrophages. These enzymes are usually down-regulated during cancer development [202], although it has also been described that they can promote tumor dissemination by a fibronectin–β1-integrin interaction and signaling in glioblastoma and ovarian cancer models [204,205].

#### 4.2.3. pH

pH is a matrix property essential for invadopodium formation. Integrins mediate cellular cues and can act as pH sensors to regulate actin protrusions [206]. α5β1, αIIβ3 and αVβ3 modify their structural activation depending on pH, and acidic stimulation promotes integrin headpiece opening and FAK-SRC signaling induction [206]. During invadopodium maturation, cortactin phosphorylation recruits Na^+^/H^+^ exchanger-1 (NHE1) to increase intracellular pH, which releases cofilin from cortactin molecules during invadopodium elongation [207]. In addition, NHE1-induced extracellular acidification increases ECM degradation and promotes tumor growth and metastasis [208]. Extracellular acidification was also observed in osteoclast podosomes. In osteoclasts, pH reduces the intracellular level of Ca^2+^ and promotes podosome formation, cell adhesion, and bone resorption [209].

#### 4.2.4. Hypoxia

Hypoxia is another microenvironment factor that induces invadosome formation. Thus, the hypoxia-inducible factor (HIF) family modulates metastases in many tumor types [210]. In melanoma cells, HIF1 and HIF2 induce SRC and FAK signaling to promote invadopodium formation and MMP2-9-dependent matrix degradation. Hypoxia also up-regulates αVβ3 expression, cell adhesion, and migration in vitronectin matrix [211]. Recently, it has been found that HIF1 induced high levels of β-PIX in invadopodia of breast cancer cell lines. β-PIX silencing reduces invadopodium formation, while its overexpression increases invadopodia [86]. Hypoxia, through HIF1, also induces many other proteins associated with invadopodia, such as the CAIX, [169]. CAIX interacts with α2/β1, α3/β1, and α6/β1 integrins as well as MMP14, and it is located in breast cancer cell mature invadopodia to induce matrix acidification and promote degradation [169].

#### 4.2.5. Protein Modification that Modulate Integrins and Invadosomes

Posttranslational modifications of intracellular, membrane, or extracellular proteins are other factors associated with invadopodium formation. Protein glycosylation is a post-transcriptional modification often related to cancer progression and metastasis [212]. The *O*-GlcNAcylation modification at serine 108 of cofilin is necessary for protein activation, and, subsequently invadopodium formation and matrix degradation in breast cancer cells [213]. Contrarily, reduced MUC1 glycosylation is associated with an increased interaction with the adaptor protein CIN85, enhancing invadopodium formation and lung metastasis of melanoma cells [214]. MUC1 is also involved in pancreatic cells migration and invasion through SRC activation mediated by αVβ5 [155]. Protein fucosylation inhibition is associated with metastatic melanomas, and fucosa treatment, or FUK overexpression in melanoma cells, inhibits invadopodia and matrix degradation [215]. Lumican is a proteoglycan that regulates collagen fibrionogenesis, and it has pro-oncogenic or anti-oncogenic properties depending on tumor type [216]. Lumican displays anti-tumoral activity by targeting α2β1, and therefore inhibiting melanoma migration [217]. In osteosarcoma, it reduces TGF-β2 activity and FAK/β1-integrin-mediated adhesion [218]. However, in colorectal cancers lumican is up-regulated and tumor cells show increased migration by actin remodeling [219]. Lumican is also up-regulated in breast and uterine cervical cancers. In prostate stroma, lumican levels increase in areas surrounding the tumors. This glycopeptide also reduces keratin 8 and 18, which are usually associated with α6β4 reduction and a less invasive phenotype. ZO-1, which interacts with α5β1 to mediate cell adhesion and lamellipodium formation, is also down-regulated in prostate cancer cells. Finally, it was also observed that lumican decreases β1-integrin and MT1-MMP expression and prostate cancer invadopodium formation to reduce tumor invasion capability [216].

### 4.3. Stromal Cells

Stromal cells are located in the surrounding microenvironment of tumor cells. Recently, new roles in tumorigenesis have emerged for these cells [220,221,222]. It has been demonstrated that stromal cells cooperate with cancer cells to facilitate metastasis of breast and other cancers in vivo [105,223,224].

Many authors have demonstrated that macrophages induce invadopodium formation in tumor cells necessary for trans-endothelial migration. During this process, macrophages, cancer, and endothelial cells form a multicellular structure called tumor microenvironment of metastasis (TMEM) that has been used as prognostic marker of metastasis in some cancer types [225]. This process depends on NOTCH signaling activation in tumor cell after cell contact [226,227], which up-regulates the expression of MENA^Inv^ and it associated with poor prognosis and invadopodium formation [226].

Although the relationship of fibroblast presence in stroma with cancer development is well demonstrated, its role in invadosome formation remains unclear. It is thought that CAFs are the major ECM producers in tumors [228], and that they use invadopodium-dependent or independent mechanisms to degrade the ECM according to the tumor type [196]. Fibroblasts express different integrins depending on extracellular matrix composition or the tissue type [228]. In fibroblasts, integrins cooperate with growth factors, such as TGF-β, in the signaling induction of tumor cell proliferation, but they also participate in matrix remodeling and promote invasion [228]. FAP is a small endopeptidase localized to lipid rafts of invadopodium rosettes in immortalized CAFs of most human epithelial tumors [229]. In melanoma cells, α3/β1 and α6/β1 function as docking proteins for seprase to form functional invadopodia to promote invasion [135,168]. FAP overexpression induces increased cell adhesion and signaling through ILK, RAC, and FAK in fibrosarcoma cell lines [230]. FAP also induces integrin downstream signaling, SRC, and PI3K, the inhibition of which reduces cell adhesion [230]. In pancreatic cancer stroma, the most abundant cells are stellate cells and CAFs, also known as myofibroblasts. CAFs contribute to tumor invasion, metastasis, and resistance to therapy [231,232], although their ablation also promotes tumor progression [233]. After phorbol ester stimulation, CAFs are able to form invadopodium structures to degrade the stroma in a CDC42 and PKC-dependent manner [234]. CAFs invadopodia and pancreatic cancer progression also require palladin expression [234]. This actin-associated protein is important for actin structure stabilization, podosome formation, and embryonic cell migration [235]. It interacts with α-actinin, which is a central element in integrin-dependent force transmission to the ECM [236]. Surprisingly, paladin knockdown increases cell force capability and induces RHO family dysfunction through actin organization and altered myosin organization [237]. Recently, it has been demonstrated that the SATB2 protein inhibits palladin-induced invadopodium formation in colorectal cancer cell lines [238]. Cao and co-workers have also shown that CAFs degrade pancreatic stroma more efficiently than epithelial pancreatic or breast cancer cells in a metalloproteinase-dependent manner [239]. However, while cancer cells form classical invadopodia, these CAFs do not present invadopodium markers in degraded areas, and they also do not show CDC42 and SRC activation [239].

Macrophages are another cell type present in tumor stroma with an important role in tumor regulation. These cells are known as tumor-associated macrophages (TAMs), and it has been reported that they can promote tumor metastasis through paracrine signals [240]. TAMs and CAFs cooperate in tumor stroma to down-regulate natural killer (NK) cell activation, and promote it in colorectal cancer development [241]. It has been described that TAMs activate their α4β1 and αLβ2 integrins to cross endothelium by interaction with VCAM and ICAM to promote tumoral accumulation [242]. Breast cancer cell lines co-cultured with TAMs increase their degradative capability and increase invadopodium formation, even in cancer cell lines with no invasive behavior. These data demonstrate that TAMs increase the invasive potential of cancer cells [243]. It is proposed that TAMs enhance CCL4 secretion to the microenvironment to induce invadopodia in tumor cells by inducing expression of MYO3A in breast cancer cell lines, which has been shown to correlate with poor prognosis [243].

## 5. Conclusions

During recent years, more and more studies have started to unveil the multifaceted roles of integrins in normal tissue remodeling and pathological processes such as tumor progression and metastasis. Crosstalk relationships with growth factor receptors as well as oncogenes have been associated with specific integrins in order to regulate cancer progression and metastasis. Numerous reports have also highlighted the importance of tumor interaction with the microenvironment and stromal factors supporting tumor growth and survival. Furthermore, deregulated integrin-mediated cell adhesion to the ECM could potentiate different stages of tumorigenesis. There is an increasing body of evidence suggesting that integrins are candidate targets for anticancer therapies, among others. In fact, effective anti-integrin therapies, such as vedolizumab, are approved for Crohn’s diseases or ulcerative colitis, and others are in advanced trials against multiple sclerosis, cardiovascular problems, or inflammatory bowel disease. Initial attempts in cancer, however, have not resulted in clinically relevant improvements, probably due to compensatory mechanisms counteracting integrin inhibition. Another possibility may be the in vitro methodological approaches of the studies, i.e., 2D environments and simple matrixes. We think that more complex environments, 3D cultures, or even more intense efforts to use in vivo models could give us a more solid base for therapy design. Apart from individual characteristics of patients, when considering integrins as possible anti-cancer targets, we should also take into account the vast integrin repertoire present in cancer and tumor stroma cells, ECM, and tumor microenvironment composition. Despite this complexity, all these factors may improve clinical stratification, advancing towards personalized medicine and allowing us to select the most effective anti-integrin combinations at appropriate stages.

## Figures and Tables

**Table 1:** Differential characteristics of each invadosome structure. N.D. (no data). * Similar to podosomes. Italic bold items indicate absent molecules that constitute a characteristic detail of the structure by their absence.

	Podosome	Linear Invadosome	Invadopodia
Cell type	Normal cells (Osteoclast, Macrophages, Fíbroblast, Dendritic cells, Smooth Muscle cells, Neurons, Endothelial and Embryonic cells)	Normal cells (Endothelial cells, Embryonic cells) Tumoral cells	Tumoral cells
Number	High (≈200)	Low (≈3–10)	Low (≈4–30)
Life-span	minutes	N.D.	hours
Width	0.5–2 μm	N.D.	0.5–2 μm
Length	0.5–2 μm	N.D.	>2 μm
Structure	Rosettes, clusters, individual	Linear structures over collagen fibers	Individual
Function	Bone resorption/Angiogenesis/Immune response/Organogenesis/Cells Interactions-fusion/Signal Mechanotrasduction	*	Cancer Metastasis
		Tumor matrix degradation	
Stimuli Inductor	Growth factor/Adhesion/Traction Force/Rigidity	Collagen Fibers	*
Signaling	SRC/ERK/PI3K/FAK/NOCH/Small GTPases	***SRC independent*** CDC42 mediated	*
Matrix anchoring	Adhesion Rings (β1/β3/α3β1/α5β1/α6β1/αVβ1 integrins/CD44)	DDR1 structure	Adhesion Rings or Not (β1 or β3/Endoglin/CD44 /DDR1)
Matrix	Mineralized matrix/Collagens/Laminin/Fibronectin/vitronectin/Basement Membrane	Collagen fibers only	*
Differential proteins detected	GRB2, Dinamin	***Absence of integrins***	NCK1, MENA^inv^, Twist, Fdg1

## References

[1] Paterson E.K., Courtneidge S.A. (2018). Invadosomes are coming: New insights into function and disease relevance. FEBS J..

[2] Lehtimaki J., Hakala M., Lappalainen P. (2017). Actin Filament Structures in Migrating Cells. Handb. Exp. Pharmacol..

[3] Murphy D.A., Courtneidge S.A. (2011). The ‘ins’ and ‘outs’ of podosomes and invadopodia: Characteristics, formation and function. Nat. Rev. Mol. Cell Biol..

[4] Porther N., Barbieri M.A. (2015). The role of endocytic Rab GTPases in regulation of growth factor signaling and the migration and invasion of tumor cells. Small GTPases.

[5] Agarwal P., Zaidel-Bar R. (2019). Principles of Actomyosin Regulation In Vivo. Trends Cell Biol..

[6] Pan L., Zhao Y., Yuan Z., Qin G. (2016). Research advances on structure and biological functions of integrins. Springerplus.

[7] Ivaska J., Thery M. (2019). Editorial overview: Cell architecture: Integrating physics and chemistry in emergent cellular properties. Curr. Opin. Cell Biol..

[8] Marsico G., Russo L., Quondamatteo F., Pandit A. (2018). Glycosylation and Integrin Regulation in Cancer. Trends Cancer.

[9] Chen W., Harbeck M.C., Zhang W., Jacobson J.R. (2013). MicroRNA regulation of integrins. Transl. Res..

[10] Moremen K.W., Tiemeyer M., Nairn A.V. (2012). Vertebrate protein glycosylation: Diversity, synthesis and function. Nat. Rev. Mol. Cell Biol..

[11] Cai X., Thinn A.M.M., Wang Z., Shan H., Zhu J. (2017). The importance of *N*-glycosylation on beta3 integrin ligand binding and conformational regulation. Sci. Rep..

[12] Hang Q., Isaji T., Hou S., Wang Y., Fukuda T., Gu J. (2017). A Key Regulator of Cell Adhesion: Identification and Characterization of Important N-Glycosylation Sites on Integrin alpha5 for Cell Migration. Mol. Cell Biol..

[13] She S., Xu B., He M., Lan X., Wang Q. (2010). Nm23-H1 suppresses hepatocarcinoma cell adhesion and migration on fibronectin by modulating glycosylation of integrin beta1. J. Exp. Clin. Cancer Res..

[14] Liu C.H., Hu R.H., Huang M.J., Lai I.R., Chen C.H., Lai H.S., Wu Y.M., Huang M.C. (2014). C1GALT1 promotes invasive phenotypes of hepatocellular carcinoma cells by modulating integrin beta1 glycosylation and activity. PLoS ONE.

[15] Radhakrishnan P., Grandgenett P.M., Mohr A.M., Bunt S.K., Yu F., Chowdhury S., Hollingsworth M.A. (2013). Expression of core 3 synthase in human pancreatic cancer cells suppresses tumor growth and metastasis. Int. J. Cancer.

[16] Tsuboi S., Hatakeyama S., Ohyama C., Fukuda M. (2012). Two opposing roles of *O*-glycans in tumor metastasis. Trends Mol. Med..

[17] Hamidi H., Ivaska J. (2018). Every step of the way: Integrins in cancer progression and metastasis. Nat. Rev. Cancer.

[18] Horton E.R., Byron A., Askari J.A., Ng D.H.J., Millon-Fremillon A., Robertson J., Koper E.J., Paul N.R., Warwood S., Knight D. (2015). Definition of a consensus integrin adhesome and its dynamics during adhesion complex assembly and disassembly. Nat. Cell Biol..

[19] Humphries J.D., Chastney M.R., Askari J.A., Humphries M.J. (2019). Signal transduction via integrin adhesion complexes. Curr. Opin. Cell Biol..

[20] Li Z., Lee H., Zhu C. (2016). Molecular mechanisms of mechanotransduction in integrin-mediated cell-matrix adhesion. Exp. Cell Res..

[21] Vicente-Manzanares M., Sanchez-Madrid F. (2018). Targeting the integrin interactome in human disease. Curr. Opin. Cell Biol..

[22] Pandolfi F., Franza L., Altamura S., Mandolini C., Cianci R., Ansari A., Kurnick J.T. (2017). Integrins: Integrating the Biology and Therapy of Cell-cell Interactions. Clin. Ther..

[23] Munger J.S., Huang X., Kawakatsu H., Griffiths M.J., Dalton S.L., Wu J., Pittet J.F., Kaminski N., Garat C., Matthay M.A. (1999). The integrin alpha v beta 6 binds and activates latent TGF beta 1: A mechanism for regulating pulmonary inflammation and fibrosis. Cell.

[24] Peng Z.W., Ikenaga N., Liu S.B., Sverdlov D.Y., Vaid K.A., Dixit R., Weinreb P.H., Violette S., Sheppard D., Schuppan D. (2016). Integrin alphavbeta6 critically regulates hepatic progenitor cell function and promotes ductular reaction, fibrosis, and tumorigenesis. Hepatology.

[25] Longmate W., DiPersio C.M. (2017). Beyond adhesion: Emerging roles for integrins in control of the tumor microenvironment. F1000Research.

[26] Yan T., Zhang A., Shi F., Chang F., Mei J., Liu Y., Zhu Y. (2018). Integrin alphavbeta3-associated DAAM1 is essential for collagen-induced invadopodia extension and cell haptotaxis in breast cancer cells. J. Biol. Chem..

[27] Stewart R.L., O’Connor K.L. (2015). Clinical significance of the integrin alpha6beta4 in human malignancies. Lab. Investig..

[28] Felding-Habermann B. (2003). Integrin adhesion receptors in tumor metastasis. Clin. Exp. Metastasis.

[29] Moreno-Layseca P., Streuli C.H. (2014). Signalling pathways linking integrins with cell cycle progression. Matrix Biol..

[30] Naci D., Aoudjit F. (2014). Alpha2beta1 integrin promotes T cell survival and migration through the concomitant activation of ERK/Mcl-1 and p38 MAPK pathways. Cell Signal..

[31] Dix C.L., Matthews H.K., Uroz M., McLaren S., Wolf L., Heatley N., Win Z., Almada P., Henriques R., Boutros M. (2018). The Role of Mitotic Cell-Substrate Adhesion Re-modeling in Animal Cell Division. Dev. Cell.

[32] Alblazi K.M., Siar C.H. (2015). Cellular protrusions--lamellipodia, filopodia, invadopodia and podosomes--and their roles in progression of orofacial tumours: Current understanding. Asian Pac. J. Cancer Prev..

[33] Kumar S., Das A., Barai A., Sen S. (2018). MMP Secretion Rate and Inter-invadopodia Spacing Collectively Govern Cancer Invasiveness. Biophys. J..

[34] David-Pfeuty T., Singer S.J. (1980). Altered distributions of the cytoskeletal proteins vinculin and alpha-actinin in cultured fibroblasts transformed by Rous sarcoma virus. Proc. Natl. Acad. Sci. USA.

[35] Di Martino J., Henriet E., Ezzoukhry Z., Goetz J.G., Moreau V., Saltel F. (2016). The microenvironment controls invadosome plasticity. J. Cell Sci..

[36] Hoshino D., Branch K.M., Weaver A.M. (2013). Signaling inputs to invadopodia and podosomes. J. Cell Sci..

[37] Juin A., Billottet C., Moreau V., Destaing O., Albiges-Rizo C., Rosenbaum J., Genot E., Saltel F. (2012). Physiological type I collagen organization induces the formation of a novel class of linear invadosomes. Mol. Biol. Cell.

[38] Veillat V., Spuul P., Daubon T., Egana I., Kramer I., Genot E. (2015). Podosomes: Multipurpose organelles?. Int. J. Biochem. Cell Biol..

[39] Eddy R.J., Weidmann M.D., Sharma V.P., Condeelis J.S. (2017). Tumor Cell Invadopodia: Invasive Protrusions that Orchestrate Metastasis. Trends Cell Biol..

[40] Kedziora K.M., Isogai T., Jalink K., Innocenti M. (2016). Invadosomes—shaping actin networks to follow mechanical cues. Front. Biosci. (Landmark Ed.).

[41] Yamaguchi H., Condeelis J. (2007). Regulation of the actin cytoskeleton in cancer cell migration and invasion. Biochim. Biophys. Acta.

[42] Gatesman A., Walker V.G., Baisden J.M., Weed S.A., Flynn D.C. (2004). Protein kinase Calpha activates c-Src and induces podosome formation via AFAP-110. Mol. Cell Biol..

[43] Varon C., Tatin F., Moreau V., Van Obberghen-Schilling E., Fernandez-Sauze S., Reuzeau E., Kramer I., Genot E. (2006). Transforming growth factor beta induces rosettes of podosomes in primary aortic endothelial cells. Mol. Cell Biol..

[44] Quintavalle M., Elia L., Condorelli G., Courtneidge S.A. (2010). MicroRNA control of podosome formation in vascular smooth muscle cells in vivo and in vitro. J. Cell Biol..

[45] Chabadel A., Banon-Rodriguez I., Cluet D., Rudkin B.B., Wehrle-Haller B., Genot E., Jurdic P., Anton I.M., Saltel F. (2007). CD44 and beta3 integrin organize two functionally distinct actin-based domains in osteoclasts. Mol. Biol. Cell.

[46] Gimona M., Buccione R. (2006). Adhesions that mediate invasion. Int. J. Biochem. Cell Biol..

[47] Juin A., Di Martino J., Leitinger B., Henriet E., Gary A.S., Paysan L., Bomo J., Baffet G., Gauthier-Rouviere C., Rosenbaum J. (2014). Discoidin domain receptor 1 controls linear invadosome formation via a Cdc42-Tuba pathway. J. Cell Biol..

[48] Yang J.C., Zhang Y., He S.J., Li M.M., Cai X.L., Wang H., Xu L.M., Cao J. (2017). TM4SF1 Promotes Metastasis of Pancreatic Cancer via Regulating the Expression of DDR1. Sci. Rep..

[49] Beaty B.T., Condeelis J. (2014). Digging a little deeper: The stages of invadopodium formation and maturation. Eur J. Cell Biol..

[50] McNiven M.A. (2013). Breaking away: Matrix remodeling from the leading edge. Trends Cell Biol..

[51] Yu C.H., Rafiq N.B., Krishnasamy A., Hartman K.L., Jones G.E., Bershadsky A.D., Sheetz M.P. (2013). Integrin-matrix clusters form podosome-like adhesions in the absence of traction forces. Cell Rep..

[52] Collin O., Na S., Chowdhury F., Hong M., Shin M.E., Wang F., Wang N. (2008). Self-organized podosomes are dynamic mechanosensors. Curr. Biol..

[53] Jacob A., Prekeris R. (2015). The regulation of MMP targeting to invadopodia during cancer metastasis. Front. Cell Dev. Biol..

[54] Albiges-Rizo C., Destaing O., Fourcade B., Planus E., Block M.R. (2009). Actin machinery and mechanosensitivity in invadopodia, podosomes and focal adhesions. J. Cell Sci..

[55] Oser M., Yamaguchi H., Mader C.C., Bravo-Cordero J.J., Arias M., Chen X., Desmarais V., van Rheenen J., Koleske A.J., Condeelis J. (2009). Cortactin regulates cofilin and N-WASp activities to control the stages of invadopodium assembly and maturation. J. Cell Biol..

[56] Yamaguchi H., Lorenz M., Kempiak S., Sarmiento C., Coniglio S., Symons M., Segall J., Eddy R., Miki H., Takenawa T. (2005). Molecular mechanisms of invadopodium formation: The role of the N-WASP-Arp2/3 complex pathway and cofilin. J. Cell Biol..

[57] Helgeson L.A., Nolen B.J. (2013). Mechanism of synergistic activation of Arp2/3 complex by cortactin and N-WASP. Elife.

[58] Linder S. (2007). The matrix corroded: Podosomes and invadopodia in extracellular matrix degradation. Trends Cell Biol..

[59] Clark E.S., Weaver A.M. (2008). A new role for cortactin in invadopodia: Regulation of protease secretion. Eur. J. Cell Biol..

[60] Antelmi E., Cardone R.A., Greco M.R., Rubino R., Di Sole F., Martino N.A., Casavola V., Carcangiu M., Moro L., Reshkin S.J. (2013). ss1 integrin binding phosphorylates ezrin at T567 to activate a lipid raft signalsome driving invadopodia activity and invasion. PLoS ONE.

[61] Hastie E.L., Sherwood D.R. (2016). A new front in cell invasion: The invadopodial membrane. Eur. J. Cell Biol..

[62] Diaz B., Shani G., Pass I., Anderson D., Quintavalle M., Courtneidge S.A. (2009). Tks5-dependent, nox-mediated generation of reactive oxygen species is necessary for invadopodia formation. Sci. Signal..

[63] Weidmann M.D., Surve C.R., Eddy R.J., Chen X., Gertler F.B., Sharma V.P., Condeelis J.S. (2016). Mena(INV) dysregulates cortactin phosphorylation to promote invadopodium maturation. Sci. Rep..

[64] Georgess D., Machuca-Gayet I., Blangy A., Jurdic P. (2014). Podosome organization drives osteoclast-mediated bone resorption. Cell Adh. Migr..

[65] Soysa N.S., Alles N. (2016). Osteoclast function and bone-resorbing activity: An overview. Biochem. Biophys. Res. Commun..

[66] Uehara S., Udagawa N., Kobayashi Y. (2018). Non-canonical Wnt signals regulate cytoskeletal remodeling in osteoclasts. Cell Mol. Life Sci..

[67] Han G., Zuo J., Holliday L.S. (2019). Specialized Roles for Actin in Osteoclasts: Unanswered Questions and Therapeutic Opportunities. Biomolecules.

[68] Bhuwania R., Cornfine S., Fang Z., Kruger M., Luna E.J., Linder S. (2012). Supervillin couples myosin-dependent contractility to podosomes and enables their turnover. J. Cell Sci..

[69] Cervero P., Wiesner C., Bouissou A., Poincloux R., Linder S. (2018). Lymphocyte-specific protein 1 regulates mechanosensory oscillation of podosomes and actin isoform-based actomyosin symmetry breaking. Nat. Commun..

[70] Schachtner H., Calaminus S.D., Sinclair A., Monypenny J., Blundell M.P., Leon C., Holyoake T.L., Thrasher A.J., Michie A.M., Vukovic M. (2013). Megakaryocytes assemble podosomes that degrade matrix and protrude through basement membrane. Blood.

[71] Cougoule C., Lastrucci C., Guiet R., Mascarau R., Meunier E., Lugo-Villarino G., Neyrolles O., Poincloux R., Maridonneau-Parini I. (2018). Podosomes, But Not the Maturation Status, Determine the Protease-Dependent 3D Migration in Human Dendritic Cells. Front. Immunol..

[72] Tarone G., Cirillo D., Giancotti F.G., Comoglio P.M., Marchisio P.C. (1985). Rous sarcoma virus-transformed fibroblasts adhere primarily at discrete protrusions of the ventral membrane called podosomes. Exp. Cell Res..

[73] Zambonin-Zallone A., Teti A., Grano M., Rubinacci A., Abbadini M., Gaboli M., Marchisio P.C. (1989). Immunocytochemical distribution of extracellular matrix receptors in human osteoclasts: A beta 3 integrin is colocalized with vinculin and talin in the podosomes of osteoclastoma giant cells. Exp. Cell Res..

[74] Seiler C., Davuluri G., Abrams J., Byfield F.J., Janmey P.A., Pack M. (2012). Smooth muscle tension induces invasive remodeling of the zebrafish intestine. PLoS Biol..

[75] Schachtner H., Calaminus S.D., Thomas S.G., Machesky L.M. (2013). Podosomes in adhesion, migration, mechanosensing and matrix remodeling. Cytoskeleton (Hoboken).

[76] Alonso F., Spuul P., Daubon T., Kramer I., Genot E. (2019). Variations on the theme of podosomes: A matter of context. Biochim. Biophys. Acta Mol. Cell Res..

[77] Spuul P., Daubon T., Pitter B., Alonso F., Fremaux I., Kramer I., Montanez E., Genot E. (2016). VEGF-A/Notch-Induced Podosomes Proteolyse Basement Membrane Collagen-IV during Retinal Sprouting Angiogenesis. Cell Rep..

[78] Xiao H., Bai X.H., Wang Y., Kim H., Mak A.S., Liu M. (2013). MEK/ERK pathway mediates PKC activation-induced recruitment of PKCzeta and MMP-9 to podosomes. J. Cell Physiol..

[79] Linder S., Nelson D., Weiss M., Aepfelbacher M. (1999). Wiskott-Aldrich syndrome protein regulates podosomes in primary human macrophages. Proc. Natl. Acad Sci. USA.

[80] Murphy D.A., Diaz B., Bromann P.A., Tsai J.H., Kawakami Y., Maurer J., Stewart R.A., Izpisua-Belmonte J.C., Courtneidge S.A. (2011). A Src-Tks5 pathway is required for neural crest cell migration during embryonic development. PLoS ONE.

[81] Cejudo-Martin P., Yuen A., Vlahovich N., Lock P., Courtneidge S.A., Diaz B. (2014). Genetic disruption of the sh3pxd2a gene reveals an essential role in mouse development and the existence of a novel isoform of tks5. PLoS ONE.

[82] Takito J., Inoue S., Nakamura M. (2018). The Sealing Zone in Osteoclasts: A Self-Organized Structure on the Bone. Int. J. Mol. Sci..

[83] Ma T., Sadashivaiah K., Madayiputhiya N., Chellaiah M.A. (2010). Regulation of sealing ring formation by L-plastin and cortactin in osteoclasts. J. Biol. Chem..

[84] Takegahara N., Kang S., Nojima S., Takamatsu H., Okuno T., Kikutani H., Toyofuku T., Kumanogoh A. (2010). Integral roles of a guanine nucleotide exchange factor, FARP2, in osteoclast podosome rearrangements. FASEB J..

[85] Kuo J.C., Han X., Hsiao C.T., Yates J.R., Waterman C.M. (2011). Analysis of the myosin-II-responsive focal adhesion proteome reveals a role for beta-Pix in negative regulation of focal adhesion maturation. Nat. Cell Biol..

[86] Md Hashim N.F., Nicholas N.S., Dart A.E., Kiriakidis S., Paleolog E., Wells C.M. (2013). Hypoxia-induced invadopodia formation: A role for beta-PIX. Open Biol..

[87] Linder S., Kopp P. (2005). Podosomes at a glance. J. Cell Sci..

[88] Gawden-Bone C., West M.A., Morrison V.L., Edgar A.J., McMillan S.J., Dill B.D., Trost M., Prescott A., Fagerholm S.C., Watts C. (2014). A crucial role for beta2 integrins in podosome formation, dynamics and Toll-like-receptor-signaled disassembly in dendritic cells. J. Cell Sci..

[89] Stylli S.S., Kaye A.H., Lock P. (2008). Invadopodia: At the cutting edge of tumour invasion. J. Clin. Neurosci..

[90] Calle Y., Burns S., Thrasher A.J., Jones G.E. (2006). The leukocyte podosome. Eur. J. Cell Biol..

[91] Erdei A., Lukacsi S., Macsik-Valent B., Nagy-Balo Z., Kurucz I., Bajtay Z. (2019). Non-identical twins: Different faces of CR3 and CR4 in myeloid and lymphoid cells of mice and men. Semin. Cell Dev. Biol..

[92] Liao Z., Kasirer-Friede A., Shattil S.J. (2017). Optogenetic interrogation of integrin alphaVbeta3 function in endothelial cells. J. Cell Sci..

[93] Obermajer N., Svajger U., Bogyo M., Jeras M., Kos J. (2008). Maturation of dendritic cells depends on proteolytic cleavage by cathepsin X. J. Leukoc. Biol.

[94] Sabri S., Foudi A., Boukour S., Franc B., Charrier S., Jandrot-Perrus M., Farndale R.W., Jalil A., Blundell M.P., Cramer E.M. (2006). Deficiency in the Wiskott-Aldrich protein induces premature proplatelet formation and platelet production in the bone marrow compartment. Blood.

[95] Schmidt S., Nakchbandi I., Ruppert R., Kawelke N., Hess M.W., Pfaller K., Jurdic P., Fassler R., Moser M. (2011). Kindlin-3-mediated signaling from multiple integrin classes is required for osteoclast-mediated bone resorption. J. Cell Biol..

[96] Cervero P., Himmel M., Kruger M., Linder S. (2012). Proteomic analysis of podosome fractions from macrophages reveals similarities to spreading initiation centres. Eur. J. Cell Biol..

[97] Ezzoukhry Z., Henriet E., Cordelieres F.P., Dupuy J.W., Maitre M., Gay N., Di-Tommaso S., Mercier L., Goetz J.G., Peter M. (2018). Combining laser capture microdissection and proteomics reveals an active translation machinery controlling invadosome formation. Nat. Commun..

[98] Pacini S., Fazzi R., Montali M., Carnicelli V., Lazzarini E., Petrini M. (2013). Specific integrin expression is associated with podosome-like structures on mesodermal progenitor cells. Stem Cells Dev..

[99] Griera M., Martin-Villar E., Banon-Rodriguez I., Blundell M.P., Jones G.E., Anton I.M., Thrasher A.J., Rodriguez-Puyol M., Calle Y. (2014). Integrin linked kinase (ILK) regulates podosome maturation and stability in dendritic cells. Int. J. Biochem. Cell Biol..

[100] Destaing O., Planus E., Bouvard D., Oddou C., Badowski C., Bossy V., Raducanu A., Fourcade B., Albiges-Rizo C., Block M.R. (2010). beta1A integrin is a master regulator of invadosome organization and function. Mol. Biol. Cell.

[101] Seano G., Primo L. (2015). Podosomes and invadopodia: Tools to breach vascular basement membrane. Cell Cycle.

[102] Seano G., Chiaverina G., Gagliardi P.A., di Blasio L., Puliafito A., Bouvard C., Sessa R., Tarone G., Sorokin L., Helley D. (2014). Endothelial podosome rosettes regulate vascular branching in tumour angiogenesis. Nat. Cell Biol..

[103] Daubon T., Spuul P., Alonso F., Fremaux I., Genot E. (2016). VEGF-A stimulates podosome-mediated collagen-IV proteolysis in microvascular endothelial cells. J. Cell Sci..

[104] Johansson M.W., Lye M.H., Barthel S.R., Duffy A.K., Annis D.S., Mosher D.F. (2004). Eosinophils adhere to vascular cell adhesion molecule-1 via podosomes. Am. J. Respir. Cell Mol. Biol..

[105] Wiesner C., Le-Cabec V., El Azzouzi K., Maridonneau-Parini I., Linder S. (2014). Podosomes in space: Macrophage migration and matrix degradation in 2D and 3D settings. Cell Adh. Migr..

[106] Van Goethem E., Guiet R., Balor S., Charriere G.M., Poincloux R., Labrousse A., Maridonneau-Parini I., Le Cabec V. (2011). Macrophage podosomes go 3D. Eur. J. Cell Biol..

[107] Lukacsi S., Nagy-Balo Z., Erdei A., Sandor N., Bajtay Z. (2017). The role of CR3 (CD11b/CD18) and CR4 (CD11c/CD18) in complement-mediated phagocytosis and podosome formation by human phagocytes. Immunol. Lett..

[108] Carman C.V., Sage P.T., Sciuto T.E., de la Fuente M.A., Geha R.S., Ochs H.D., Dvorak H.F., Dvorak A.M., Springer T.A. (2007). Transcellular diapedesis is initiated by invasive podosomes. Immunity.

[109] Teijeira A., Garasa S., Pelaez R., Azpilikueta A., Ochoa C., Marre D., Rodrigues M., Alfaro C., Auba C., Valitutti S. (2013). Lymphatic endothelium forms integrin-engaging 3D structures during DC transit across inflamed lymphatic vessels. J. Investig. Dermatol..

[110] Mennens S.F.B., Bolomini-Vittori M., Weiden J., Joosten B., Cambi A., van den Dries K. (2017). Substrate stiffness influences phenotype and function of human antigen-presenting dendritic cells. Sci. Rep..

[111] Johansson M.W., Annis D.S., Mosher D.F. (2013). alpha(M)beta(2) integrin-mediated adhesion and motility of IL-5-stimulated eosinophils on periostin. Am. J. Respir. Cell Mol. Biol..

[112] Duong L.T., Rodan G.A. (1998). Integrin-mediated signaling in the regulation of osteoclast adhesion and activation. Front. Biosci..

[113] Ponceau A., Albiges-Rizo C., Colin-Aronovicz Y., Destaing O., Lecomte M.C. (2015). alphaII-spectrin regulates invadosome stability and extracellular matrix degradation. PLoS ONE.

[114] Luxenburg C., Winograd-Katz S., Addadi L., Geiger B. (2012). Involvement of actin polymerization in podosome dynamics. J. Cell Sci..

[115] Zou W., Kitaura H., Reeve J., Long F., Tybulewicz V.L., Shattil S.J., Ginsberg M.H., Ross F.P., Teitelbaum S.L. (2007). Syk, c-Src, the alphavbeta3 integrin, and ITAM immunoreceptors, in concert, regulate osteoclastic bone resorption. J. Cell Biol..

[116] Yaroslavskiy B.B., Zhang Y., Kalla S.E., Garcia Palacios V., Sharrow A.C., Li Y., Zaidi M., Wu C., Blair H.C. (2005). NO-dependent osteoclast motility: Reliance on cGMP-dependent protein kinase I and VASP. J. Cell Sci..

[117] Chellaiah M.A. (2006). Regulation of podosomes by integrin alphavbeta3 and Rho GTPase-facilitated phosphoinositide signaling. Eur. J. Cell Biol..

[118] Spinardi L., Rietdorf J., Nitsch L., Bono M., Tacchetti C., Way M., Marchisio P.C. (2004). A dynamic podosome-like structure of epithelial cells. Exp. Cell Res..

[119] Takkunen M., Hukkanen M., Liljestrom M., Grenman R., Virtanen I. (2010). Podosome-like structures of non-invasive carcinoma cells are replaced in epithelial-mesenchymal transition by actin comet-embedded invadopodia. J. Cell Mol. Med..

[120] Markwell S.M., Gatesman Ammer A.G., Interval E.T., Allen J.L., Papenberg B.W., Hames R.A., Castano J.E., Schafer D.A., Weed S.A. (2019). Cortactin Phosphorylation by Casein Kinase 2 Regulates Actin-Related Protein 2/3 Complex Activity, Invadopodia Function and Tumor Cell Invasion. Mol. Cancer Res..

[121] Meirson T., Gil-Henn H. (2018). Targeting invadopodia for blocking breast cancer metastasis. Drug Resist. Updat..

[122] Gurski L.A., Knowles L.M., Basse P.H., Maranchie J.K., Watkins S.C., Pilch J. (2015). Relocation of CLIC1 promotes tumor cell invasion and colonization of fibrin. Mol. Cancer Res..

[123] Bergamaschi A., Tagliabue E., Sorlie T., Naume B., Triulzi T., Orlandi R., Russnes H.G., Nesland J.M., Tammi R., Auvinen P. (2008). Extracellular matrix signature identifies breast cancer subgroups with different clinical outcome. J. Pathol..

[124] Leong H.S., Robertson A.E., Stoletov K., Leith S.J., Chin C.A., Chien A.E., Hague M.N., Ablack A., Carmine-Simmen K., McPherson V.A. (2014). Invadopodia are required for cancer cell extravasation and are a therapeutic target for metastasis. Cell Rep..

[125] Tokui N., Yoneyama M.S., Hatakeyama S., Yamamoto H., Koie T., Saitoh H., Yamaya K., Funyu T., Nakamura T., Ohyama C. (2014). Extravasation during bladder cancer metastasis requires cortactinmediated invadopodia formation. Mol. Med. Rep..

[126] Blouw B., Patel M., Iizuka S., Abdullah C., You W.K., Huang X., Li J.L., Diaz B., Stallcup W.B., Courtneidge S.A. (2015). The invadopodia scaffold protein Tks5 is required for the growth of human breast cancer cells in vitro and in vivo. PLoS ONE.

[127] Iizuka S., Abdullah C., Buschman M.D., Diaz B., Courtneidge S.A. (2016). The role of Tks adaptor proteins in invadopodia formation, growth and metastasis of melanoma. Oncotarget.

[128] Blouw B., Seals D.F., Pass I., Diaz B., Courtneidge S.A. (2008). A role for the podosome/invadopodia scaffold protein Tks5 in tumor growth in vivo. Eur. J. Cell Biol..

[129] Gligorijevic B., Wyckoff J., Yamaguchi H., Wang Y., Roussos E.T., Condeelis J. (2012). N-WASP-mediated invadopodium formation is involved in intravasation and lung metastasis of mammary tumors. J. Cell Sci..

[130] Alexander N.R., Branch K.M., Parekh A., Clark E.S., Iwueke I.C., Guelcher S.A., Weaver A.M. (2008). Extracellular matrix rigidity promotes invadopodia activity. Curr. Biol..

[131] Gil-Henn H., Destaing O., Sims N.A., Aoki K., Alles N., Neff L., Sanjay A., Bruzzaniti A., De Camilli P., Baron R. (2007). Defective microtubule-dependent podosome organization in osteoclasts leads to increased bone density in Pyk2(−/−) mice. J. Cell Biol..

[132] Genna A., Lapetina S., Lukic N., Twafra S., Meirson T., Sharma V.P., Condeelis J.S., Gil-Henn H. (2018). Pyk2 and FAK differentially regulate invadopodia formation and function in breast cancer cells. J. Cell Biol..

[133] MacGrath S.M., Koleske A.J. (2012). Cortactin in cell migration and cancer at a glance. J. Cell Sci..

[134] Ayala I., Baldassarre M., Giacchetti G., Caldieri G., Tete S., Luini A., Buccione R. (2008). Multiple regulatory inputs converge on cortactin to control invadopodia biogenesis and extracellular matrix degradation. J. Cell Sci..

[135] Mueller S.C., Ghersi G., Akiyama S.K., Sang Q.X., Howard L., Pineiro-Sanchez M., Nakahara H., Yeh Y., Chen W.T. (1999). A novel protease-docking function of integrin at invadopodia. J. Biol. Chem..

[136] Branch K.M., Hoshino D., Weaver A.M. (2012). Adhesion rings surround invadopodia and promote maturation. Biol. Open.

[137] Knowles L.M., Gurski L.A., Engel C., Gnarra J.R., Maranchie J.K., Pilch J. (2013). Integrin alphavbeta3 and fibronectin upregulate Slug in cancer cells to promote clot invasion and metastasis. Cancer Res..

[138] Pelaez R., Morales X., Salvo E., Garasa S., Ortiz de Solorzano C., Martinez A., Larrayoz I.M., Rouzaut A. (2017). β3 integrin expression is required for invadopodia-mediated ECM degradation in lung carcinoma cells. PLoS ONE.

[139] Milone M.R., Pucci B., Colangelo T., Lombardi R., Iannelli F., Colantuoni V., Sabatino L., Budillon A. (2016). Proteomic characterization of peroxisome proliferator-activated receptor-gamma (PPARgamma) overexpressing or silenced colorectal cancer cells unveils a novel protein network associated with an aggressive phenotype. Mol. Oncol.

[140] Mallawaaratchy D.M., Hallal S., Russell B., Ly L., Ebrahimkhani S., Wei H., Christopherson R.I., Buckland M.E., Kaufman K.L. (2017). Comprehensive proteome profiling of glioblastoma-derived extracellular vesicles identifies markers for more aggressive disease. J. Neurooncol..

[141] Havrylov S., Park M. (2015). MS/MS-based strategies for proteomic profiling of invasive cell structures. Proteomics.

[142] Shen B., Delaney M.K., Du X. (2012). Inside-out, outside-in, and inside-outside-in: G protein signaling in integrin-mediated cell adhesion, spreading, and retraction. Curr. Opin. Cell Biol..

[143] Zent J., Guo L.W. (2018). Signaling Mechanisms of Myofibroblastic Activation: Outside-in and Inside-Out. Cell Physiol. Biochem..

[144] Yamaguchi H., Takeo Y., Yoshida S., Kouchi Z., Nakamura Y., Fukami K. (2009). Lipid rafts and caveolin-1 are required for invadopodia formation and extracellular matrix degradation by human breast cancer cells. Cancer Res..

[145] Salvo E., Garasa S., Dotor J., Morales X., Pelaez R., Altevogt P., Rouzaut A. (2014). Combined targeting of TGF-beta1 and integrin beta3 impairs lymph node metastasis in a mouse model of non-small-cell lung cancer. Mol. Cancer.

[146] Ghatak S., Misra S., Moreno-Rodrigue R.A., Hascall V.C., Leone G.W., Markwald R.R. (2019). Periostin/beta1integrin interaction regulates p21-activated kinases in valvular interstitial cell survival and in actin cytoskeleton reorganization. Biochim. Biophys. Acta Gen. Subj..

[147] Mainiero F., Colombara M., Antonini V., Strippoli R., Merola M., Poffe O., Tridente G., Ramarli D. (2003). p38 MAPK is a critical regulator of the constitutive and the beta4 integrin-regulated expression of IL-6 in human normal thymic epithelial cells. Eur. J. Immunol..

[148] Sundberg-Smith L.J., Doherty J.T., Mack C.P., Taylor J.M. (2005). Adhesion stimulates direct PAK1/ERK2 association and leads to ERK-dependent PAK1 Thr212 phosphorylation. J. Biol. Chem..

[149] Maziveyi M., Dong S., Baranwal S., Alahari S.K. (2018). Nischarin regulates focal adhesion and Invadopodia formation in breast cancer cells. Mol. Cancer.

[150] Gagliardi P.A., di Blasio L., Primo L. (2015). PDK1: A signaling hub for cell migration and tumor invasion. Biochim. Biophys. Acta.

[151] Williams K.C., Cepeda M.A., Javed S., Searle K., Parkins K.M., Makela A.V., Hamilton A.M., Soukhtehzari S., Kim Y., Tuck A.B. (2019). Invadopodia are chemosensing protrusions that guide cancer cell extravasation to promote brain tropism in metastasis. Oncogene.

[152] Haidari M., Zhang W., Caivano A., Chen Z., Ganjehei L., Mortazavi A., Stroud C., Woodside D.G., Willerson J.T., Dixon R.A. (2012). Integrin alpha2beta1 mediates tyrosine phosphorylation of vascular endothelial cadherin induced by invasive breast cancer cells. J. Biol. Chem..

[153] Gasparski A.N., Ozarkar S., Beningo K.A. (2017). Transient mechanical strain promotes the maturation of invadopodia and enhances cancer cell invasion in vitro. J. Cell Sci..

[154] Beaty B.T., Sharma V.P., Bravo-Cordero J.J., Simpson M.A., Eddy R.J., Koleske A.J., Condeelis J. (2013). β1 integrin regulates Arg to promote invadopodial maturation and matrix degradation. Mol. Biol. Cell.

[155] Lau S.K., Shields D.J., Murphy E.A., Desgrosellier J.S., Anand S., Huang M., Kato S., Lim S.T., Weis S.M., Stupack D.G. (2012). EGFR-mediated carcinoma cell metastasis mediated by integrin alphavbeta5 depends on activation of c-Src and cleavage of MUC1. PLoS ONE.

[156] Liu J., Yue P., Artym V.V., Mueller S.C., Guo W. (2009). The role of the exocyst in matrix metalloproteinase secretion and actin dynamics during tumor cell invadopodia formation. Mol. Biol. Cell.

[157] Hoshino D., Kirkbride K.C., Costello K., Clark E.S., Sinha S., Grega-Larson N., Tyska M.J., Weaver A.M. (2013). Exosome secretion is enhanced by invadopodia and drives invasive behavior. Cell Rep..

[158] Hoshino A., Costa-Silva B., Shen T.L., Rodrigues G., Hashimoto A., Tesic Mark M., Molina H., Kohsaka S., Di Giannatale A., Ceder S. (2015). Tumour exosome integrins determine organotropic metastasis. Nature.

[159] Maia J., Caja S., Strano Moraes M.C., Couto N., Costa-Silva B. (2018). Exosome-Based Cell-Cell Communication in the Tumor Microenvironment. Front. Cell Dev. Biol..

[160] Valcz G., Buzas E.I., Szallasi Z., Kalmar A., Krenacs T., Tulassay Z., Igaz P., Molnar B. (2018). Perspective: Bidirectional exosomal transport between cancer stem cells and their fibroblast-rich microenvironment during metastasis formation. NPJ Breast Cancer.

[161] Desgrosellier J.S., Cheresh D.A. (2010). Integrins in cancer: Biological implications and therapeutic opportunities. Nat. Rev. Cancer.

[162] Hersey P., Sosman J., O’Day S., Richards J., Bedikian A., Gonzalez R., Sharfman W., Weber R., Logan T., Buzoianu M. (2010). A randomized phase 2 study of etaracizumab, a monoclonal antibody against integrin alpha(v)beta(3), + or − dacarbazine in patients with stage IV metastatic melanoma. Cancer.

[163] Mallawaaratchy D.M., Buckland M.E., McDonald K.L., Li C.C., Ly L., Sykes E.K., Christopherson R.I., Kaufman K.L. (2015). Membrane proteome analysis of glioblastoma cell invasion. J. Neuropathol. Exp. Neurol..

[164] Mullamitha S.A., Ton N.C., Parker G.J., Jackson A., Julyan P.J., Roberts C., Buonaccorsi G.A., Watson Y., Davies K., Cheung S. (2007). Phase I evaluation of a fully human anti-alphav integrin monoclonal antibody (CNTO 95) in patients with advanced solid tumors. Clin. Cancer Res..

[165] Mateo J., Berlin J., de Bono J.S., Cohen R.B., Keedy V., Mugundu G., Zhang L., Abbattista A., Davis C., Gallo Stampino C. (2014). A first-in-human study of the anti-alpha5beta1 integrin monoclonal antibody PF-04605412 administered intravenously to patients with advanced solid tumors. Cancer Chemother. Pharmacol..

[166] Cianfrocca M.E., Kimmel K.A., Gallo J., Cardoso T., Brown M.M., Hudes G., Lewis N., Weiner L., Lam G.N., Brown S.C. (2006). Phase 1 trial of the antiangiogenic peptide ATN-161 (Ac-PHSCN-NH(2)), a beta integrin antagonist, in patients with solid tumours. Br. J. Cancer.

[167] Seftor R.E., Seftor E.A., Stetler-Stevenson W.G., Hendrix M.J. (1993). The 72 kDa type IV collagenase is modulated via differential expression of alpha v beta 3 and alpha 5 beta 1 integrins during human melanoma cell invasion. Cancer Res..

[168] Nakahara H., Nomizu M., Akiyama S.K., Yamada Y., Yeh Y., Chen W.T. (1996). A mechanism for regulation of melanoma invasion. Ligation of alpha6beta1 integrin by laminin G peptides. J. Biol. Chem..

[169] Swayampakula M., McDonald P.C., Vallejo M., Coyaud E., Chafe S.C., Westerback A., Venkateswaran G., Shankar J., Gao G., Laurent E.M.N. (2017). The interactome of metabolic enzyme carbonic anhydrase IX reveals novel roles in tumor cell migration and invadopodia/MMP14-mediated invasion. Oncogene.

[170] Siqueira A.S., Pinto M.P., Cruz M.C., Smuczek B., Cruz K.S., Barbuto J.A., Hoshino D., Weaver A.M., Freitas V.M., Jaeger R.G. (2016). Laminin-111 peptide C16 regulates invadopodia activity of malignant cells through beta1 integrin, Src and ERK 1/2. Oncotarget.

[171] Artym V.V., Swatkoski S., Matsumoto K., Campbell C.B., Petrie R.J., Dimitriadis E.K., Li X., Mueller S.C., Bugge T.H., Gucek M. (2015). Dense fibrillar collagen is a potent inducer of invadopodia via a specific signaling network. J. Cell Biol..

[172] Seftor R.E., Seftor E.A., Gehlsen K.R., Stetler-Stevenson W.G., Brown P.D., Ruoslahti E., Hendrix M.J. (1992). Role of the alpha v beta 3 integrin in human melanoma cell invasion. Proc. Natl. Acad Sci. USA.

[173] Deryugina E.I., Ratnikov B., Monosov E., Postnova T.I., DiScipio R., Smith J.W., Strongin A.Y. (2001). MT1-MMP initiates activation of pro-MMP-2 and integrin alphavbeta3 promotes maturation of MMP-2 in breast carcinoma cells. Exp. Cell Res..

[174] Brooks P.C., Stromblad S., Sanders L.C., von Schalscha T.L., Aimes R.T., Stetler-Stevenson W.G., Quigley J.P., Cheresh D.A. (1996). Localization of matrix metalloproteinase MMP-2 to the surface of invasive cells by interaction with integrin alpha v beta 3. Cell.

[175] Malik G., Knowles L.M., Dhir R., Xu S., Yang S., Ruoslahti E., Pilch J. (2010). Plasma fibronectin promotes lung metastasis by contributions to fibrin clots and tumor cell invasion. Cancer Res..

[176] Cheerathodi M., Avci N.G., Guerrero P.A., Tang L.K., Popp J., Morales J.E., Chen Z., Carnero A., Lang F.F., Ballif B.A. (2016). The Cytoskeletal Adapter Protein Spinophilin Regulates Invadopodia Dynamics and Tumor Cell Invasion in Glioblastoma. Mol. Cancer Res..

[177] Jannuzi A.L., Bunch T.A., West R.F., Brower D.L. (2004). Identification of integrin beta subunit mutations that alter heterodimer function in situ. Mol. Biol. Cell.

[178] Baranwal S., Wang Y., Rathinam R., Lee J., Jin L., McGoey R., Pylayeva Y., Giancotti F., Blobe G.C., Alahari S.K. (2011). Molecular characterization of the tumor-suppressive function of nischarin in breast cancer. J. Natl. Cancer Inst..

[179] Parekh A., Ruppender N.S., Branch K.M., Sewell-Loftin M.K., Lin J., Boyer P.D., Candiello J.E., Merryman W.D., Guelcher S.A., Weaver A.M. (2011). Sensing and modulation of invadopodia across a wide range of rigidities. Biophys. J..

[180] Yurchenco P.D., Amenta P.S., Patton B.L. (2004). Basement membrane assembly, stability and activities observed through a developmental lens. Matrix Biol..

[181] Hynes R.O. (2014). Stretching the boundaries of extracellular matrix research. Nat. Rev. Mol. Cell Biol..

[182] Liu S., Yamashita H., Weidow B., Weaver A.M., Quaranta V. (2010). Laminin-332-beta1 integrin interactions negatively regulate invadopodia. J. Cell Physiol..

[183] van Kempen L.C., Rijntjes J., Mamor-Cornelissen I., Vincent-Naulleau S., Gerritsen M.J., Ruiter D.J., van Dijk M.C., Geffrotin C., van Muijen G.N. (2008). Type I collagen expression contributes to angiogenesis and the development of deeply invasive cutaneous melanoma. Int. J. Cancer.

[184] Conklin M.W., Keely P.J. (2012). Why the stroma matters in breast cancer: Insights into breast cancer patient outcomes through the examination of stromal biomarkers. Cell Adh. Migr..

[185] Miyake M., Hori S., Morizawa Y., Tatsumi Y., Toritsuka M., Ohnishi S., Shimada K., Furuya H., Khadka V.S., Deng Y. (2017). Collagen type IV alpha 1 (COL4A1) and collagen type XIII alpha 1 (COL13A1) produced in cancer cells promote tumor budding at the invasion front in human urothelial carcinoma of the bladder. Oncotarget.

[186] Adiguzel E., Hou G., Sabatini P.J., Bendeck M.P. (2013). Type VIII collagen signals via beta1 integrin and RhoA to regulate MMP-2 expression and smooth muscle cell migration. Matrix Biol..

[187] Labernadie A., Thibault C., Vieu C., Maridonneau-Parini I., Charriere G.M. (2010). Dynamics of podosome stiffness revealed by atomic force microscopy. Proc. Natl. Acad Sci. USA.

[188] Liu J., Tan Y., Zhang H., Zhang Y., Xu P., Chen J., Poh Y.C., Tang K., Wang N., Huang B. (2012). Soft fibrin gels promote selection and growth of tumorigenic cells. Nat. Mater..

[189] Ponce M.L., Nomizu M., Kleinman H.K. (2001). An angiogenic laminin site and its antagonist bind through the alpha(v)beta3 and alpha5beta1 integrins. FASEB J..

[190] Nascimento C.F., de Siqueira A.S., Pinheiro J.J., Freitas V.M., Jaeger R.G. (2011). Laminin-111 derived peptides AG73 and C16 regulate invadopodia activity of a human adenoid cystic carcinoma cell line. Exp. Cell Res..

[191] Guess C.M., Lafleur B.J., Weidow B.L., Quaranta V. (2009). A decreased ratio of laminin-332 beta3 to gamma2 subunit mRNA is associated with poor prognosis in colon cancer. Cancer Epidemiol. Biomarkers Prev..

[192] Rousselle P., Lunstrum G.P., Keene D.R., Burgeson R.E. (1991). Kalinin: An epithelium-specific basement membrane adhesion molecule that is a component of anchoring filaments. J. Cell Biol..

[193] Jerrell R.J., Parekh A. (2016). Matrix rigidity differentially regulates invadopodia activity through ROCK1 and ROCK2. Biomaterials.

[194] Provenzano P.P., Inman D.R., Eliceiri K.W., Knittel J.G., Yan L., Rueden C.T., White J.G., Keely P.J. (2008). Collagen density promotes mammary tumor initiation and progression. BMC Med..

[195] Boyd N.F., Martin L.J., Rommens J.M., Paterson A.D., Minkin S., Yaffe M.J., Stone J., Hopper J.L. (2009). Mammographic density: A heritable risk factor for breast cancer. Methods Mol. Biol..

[196] Parekh A., Weaver A.M. (2016). Regulation of invadopodia by mechanical signaling. Exp. Cell Res..

[197] Miles F.L., Sikes R.A. (2014). Insidious changes in stromal matrix fuel cancer progression. Mol. Cancer Res..

[198] Jerrell R.J., Parekh A. (2014). Cellular traction stresses mediate extracellular matrix degradation by invadopodia. Acta Biomater..

[199] Levental K.R., Yu H., Kass L., Lakins J.N., Egeblad M., Erler J.T., Fong S.F., Csiszar K., Giaccia A., Weninger W. (2009). Matrix crosslinking forces tumor progression by enhancing integrin signaling. Cell.

[200] Engler A., Bacakova L., Newman C., Hategan A., Griffin M., Discher D. (2004). Substrate compliance versus ligand density in cell on gel responses. Biophys. J..

[201] Roca-Cusachs P., Iskratsch T., Sheetz M.P. (2012). Finding the weakest link: Exploring integrin-mediated mechanical molecular pathways. J. Cell Sci..

[202] Enderling H., Alexander N.R., Clark E.S., Branch K.M., Estrada L., Crooke C., Jourquin J., Lobdell N., Zaman M.H., Guelcher S.A. (2008). Dependence of invadopodia function on collagen fiber spacing and cross-linking: Computational modeling and experimental evidence. Biophys. J..

[203] Burgstaller G., Gimona M. (2005). Podosome-mediated matrix resorption and cell motility in vascular smooth muscle cells. Am. J. Physiol. Heart Circ. Physiol..

[204] Yuan L., Siegel M., Choi K., Khosla C., Miller C.R., Jackson E.N., Piwnica-Worms D., Rich K.M. (2007). Transglutaminase 2 inhibitor, KCC009, disrupts fibronectin assembly in the extracellular matrix and sensitizes orthotopic glioblastomas to chemotherapy. Oncogene.

[205] Satpathy M., Cao L., Pincheira R., Emerson R., Bigsby R., Nakshatri H., Matei D. (2007). Enhanced peritoneal ovarian tumor dissemination by tissue transglutaminase. Cancer Res..

[206] Li S., Xiong N., Peng Y., Tang K., Bai H., Lv X., Jiang Y., Qin X., Yang H., Wu C. (2018). Acidic pHe regulates cytoskeletal dynamics through conformational integrin beta1 activation and promotes membrane protrusion. Biochim. Biophys. Acta Mol. Basis. Dis..

[207] Magalhaes M.A., Larson D.R., Mader C.C., Bravo-Cordero J.J., Gil-Henn H., Oser M., Chen X., Koleske A.J., Condeelis J. (2011). Cortactin phosphorylation regulates cell invasion through a pH-dependent pathway. J. Cell Biol..

[208] Busco G., Cardone R.A., Greco M.R., Bellizzi A., Colella M., Antelmi E., Mancini M.T., Dell’Aquila M.E., Casavola V., Paradiso A. (2010). NHE1 promotes invadopodial ECM proteolysis through acidification of the peri-invadopodial space. FASEB J..

[209] Teti A., Blair H.C., Schlesinger P., Grano M., Zambonin-Zallone A., Kahn A.J., Teitelbaum S.L., Hruska K.A. (1989). Extracellular protons acidify osteoclasts, reduce cytosolic calcium, and promote expression of cell-matrix attachment structures. J. Clin. Investig..

[210] Rankin E.B., Giaccia A.J. (2016). Hypoxic control of metastasis. Science.

[211] Cowden Dahl K.D., Robertson S.E., Weaver V.M., Simon M.C. (2005). Hypoxia-inducible factor regulates alphavbeta3 integrin cell surface expression. Mol. Biol. Cell.

[212] Munkley J., Elliott D.J. (2016). Hallmarks of glycosylation in cancer. Oncotarget.

[213] Huang X., Pan Q., Sun D., Chen W., Shen A., Huang M., Ding J., Geng M. (2013). *O*-GlcNAcylation of cofilin promotes breast cancer cell invasion. J. Biol. Chem..

[214] Cascio S., Farkas A.M., Hughey R.P., Finn O.J. (2013). Altered glycosylation of MUC1 influences its association with CIN85: The role of this novel complex in cancer cell invasion and migration. Oncotarget.

[215] Keeley T., Lin S., Lester D.K., Lau E.K., Yang S. (2018). The fucose salvage pathway inhibits invadopodia formation and extracellular matrix degradation in melanoma cells. PLoS ONE.

[216] Coulson-Thomas V.J., Coulson-Thomas Y.M., Gesteira T.F., Andrade de Paula C.A., Carneiro C.R., Ortiz V., Toma L., Kao W.W., Nader H.B. (2013). Lumican expression, localization and antitumor activity in prostate cancer. Exp. Cell Res..

[217] Zeltz C., Brezillon S., Kapyla J., Eble J.A., Bobichon H., Terryn C., Perreau C., Franz C.M., Heino J., Maquart F.X. (2010). Lumican inhibits cell migration through alpha2beta1 integrin. Exp. Cell Res..

[218] Nikitovic D., Chalkiadaki G., Berdiaki A., Aggelidakis J., Katonis P., Karamanos N.K., Tzanakakis G.N. (2011). Lumican regulates osteosarcoma cell adhesion by modulating TGFbeta2 activity. Int. J. Biochem. Cell Biol..

[219] Radwanska A., Litwin M., Nowak D., Baczynska D., Wegrowski Y., Maquart F.X., Malicka-Blaszkiewicz M. (2012). Overexpression of lumican affects the migration of human colon cancer cells through up-regulation of gelsolin and filamentous actin reorganization. Exp. Cell Res..

[220] Larrayoz I.M., Martinez-Herrero S., Garcia-Sanmartin J., Ochoa-Callejero L., Martinez A. (2014). Adrenomedullin and tumour microenvironment. J. Transl. Med..

[221] Thomas D., Radhakrishnan P. (2019). Tumor-stromal crosstalk in pancreatic cancer and tissue fibrosis. Mol. Cancer.

[222] Adamo A., Dal Collo G., Bazzoni R., Krampera M. (2019). Role of mesenchymal stromal cell-derived extracellular vesicles in tumour microenvironment. Biochim. Biophys. Acta Rev. Cancer.

[223] Wyckoff J.B., Wang Y., Lin E.Y., Li J.F., Goswami S., Stanley E.R., Segall J.E., Pollard J.W., Condeelis J. (2007). Direct visualization of macrophage-assisted tumor cell intravasation in mammary tumors. Cancer Res..

[224] Harney A.S., Arwert E.N., Entenberg D., Wang Y., Guo P., Qian B.Z., Oktay M.H., Pollard J.W., Jones J.G., Condeelis J.S. (2015). Real-Time Imaging Reveals Local, Transient Vascular Permeability, and Tumor Cell Intravasation Stimulated by TIE2hi Macrophage-Derived VEGFA. Cancer Discov..

[225] Rohan T.E., Xue X., Lin H.M., D’Alfonso T.M., Ginter P.S., Oktay M.H., Robinson B.D., Ginsberg M., Gertler F.B., Glass A.G. (2014). Tumor microenvironment of metastasis and risk of distant metastasis of breast cancer. J. Natl. Cancer Inst..

[226] Pignatelli J., Bravo-Cordero J.J., Roh-Johnson M., Gandhi S.J., Wang Y., Chen X., Eddy R.J., Xue A., Singer R.H., Hodgson L. (2016). Macrophage-dependent tumor cell transendothelial migration is mediated by Notch1/Mena(INV)-initiated invadopodium formation. Sci. Rep..

[227] Diaz B., Yuen A., Iizuka S., Higashiyama S., Courtneidge S.A. (2013). Notch increases the shedding of HB-EGF by ADAM12 to potentiate invadopodia formation in hypoxia. J. Cell Biol..

[228] Multhaupt H.A., Leitinger B., Gullberg D., Couchman J.R. (2016). Extracellular matrix component signaling in cancer. Adv. Drug Deliv. Rev..

[229] Knopf J.D., Tholen S., Koczorowska M.M., De Wever O., Biniossek M.L., Schilling O. (2015). The stromal cell-surface protease fibroblast activation protein-alpha localizes to lipid rafts and is recruited to invadopodia. Biochim. Biophys. Acta.

[230] Baird S.K., Allan L., Renner C., Scott F.E., Scott A.M. (2015). Fibroblast activation protein increases metastatic potential of fibrosarcoma line HT1080 through upregulation of integrin-mediated signaling pathways. Clin. Exp. Metastasis.

[231] Orimo A., Gupta P.B., Sgroi D.C., Arenzana-Seisdedos F., Delaunay T., Naeem R., Carey V.J., Richardson A.L., Weinberg R.A. (2005). Stromal fibroblasts present in invasive human breast carcinomas promote tumor growth and angiogenesis through elevated SDF-1/CXCL12 secretion. Cell.

[232] Gaggioli C., Hooper S., Hidalgo-Carcedo C., Grosse R., Marshall J.F., Harrington K., Sahai E. (2007). Fibroblast-led collective invasion of carcinoma cells with differing roles for RhoGTPases in leading and following cells. Nat. Cell Biol..

[233] Ozdemir B.C., Pentcheva-Hoang T., Carstens J.L., Zheng X., Wu C.C., Simpson T.R., Laklai H., Sugimoto H., Kahlert C., Novitskiy S.V. (2014). Depletion of carcinoma-associated fibroblasts and fibrosis induces immunosuppression and accelerates pancreas cancer with reduced survival. Cancer Cell..

[234] Goicoechea S.M., Garcia-Mata R., Staub J., Valdivia A., Sharek L., McCulloch C.G., Hwang R.F., Urrutia R., Yeh J.J., Kim H.J. (2014). Palladin promotes invasion of pancreatic cancer cells by enhancing invadopodia formation in cancer-associated fibroblasts. Oncogene.

[235] Goicoechea S.M., Bednarski B., Garcia-Mata R., Prentice-Dunn H., Kim H.J., Otey C.A. (2009). Palladin contributes to invasive motility in human breast cancer cells. Oncogene.

[236] Roca-Cusachs P., del Rio A., Puklin-Faucher E., Gauthier N.C., Biais N., Sheetz M.P. (2013). Integrin-dependent force transmission to the extracellular matrix by alpha-actinin triggers adhesion maturation. Proc. Natl. Acad. Sci. USA.

[237] Azatov M., Goicoechea S.M., Otey C.A., Upadhyaya A. (2016). The actin crosslinking protein palladin modulates force generation and mechanosensitivity of tumor associated fibroblasts. Sci. Rep..

[238] Mansour M.A., Asano E., Hyodo T., Akter K.A., Takahashi M., Hamaguchi M., Senga T. (2015). Special AT-rich sequence-binding protein 2 suppresses invadopodia formation in HCT116 cells via palladin inhibition. Exp. Cell Res..

[239] Cao H., Eppinga R.D., Razidlo G.L., Krueger E.W., Chen J., Qiang L., McNiven M.A. (2016). Stromal fibroblasts facilitate cancer cell invasion by a novel invadopodia-independent matrix degradation process. Oncogene.

[240] Joyce J.A., Pollard J.W. (2009). Microenvironmental regulation of metastasis. Nat. Rev. Cancer.

[241] Zhang R., Qi F., Zhao F., Li G., Shao S., Zhang X., Yuan L., Feng Y. (2019). Cancer-associated fibroblasts enhance tumor-associated macrophages enrichment and suppress NK cells function in colorectal cancer. Cell Death Dis..

[242] Pathria P., Louis T.L., Varner J.A. (2019). Targeting Tumor-Associated Macrophages in Cancer. Trends Immunol..

[243] Baghel K.S., Tewari B.N., Shrivastava R., Malik S.A., Lone M.U., Jain N.K., Tripathi C., Kanchan R.K., Dixit S., Singh K. (2016). Macrophages promote matrix protrusive and invasive function of breast cancer cells via MIP-1beta dependent upregulation of MYO3A gene in breast cancer cells. Oncoimmunology.

